# Temporal Correlation Mechanisms and Their Role in Feature Selection: A Single-Unit Study in Primate Somatosensory Cortex

**DOI:** 10.1371/journal.pbio.1002004

**Published:** 2014-11-25

**Authors:** Manuel Gomez-Ramirez, Natalie K. Trzcinski, Stefan Mihalas, Ernst Niebur, Steven S. Hsiao

**Affiliations:** 1 The Zanvyl Krieger Mind/Brain Institute, The Johns Hopkins University, Baltimore, Maryland, United States of America; 2 The Solomon H. Snyder Department of Neuroscience, The Johns Hopkins School of Medicine, Baltimore, Maryland, United States of America; Yeshiva University Albert Einstein College of Medicine, United States of America

## Abstract

Studies in vision show that attention enhances the firing rates of cells when it is directed towards their preferred stimulus feature. However, it is unknown whether other sensory systems employ this mechanism to mediate feature selection within their modalities. Moreover, whether feature-based attention modulates the correlated activity of a population is unclear. Indeed, temporal correlation codes such as spike-synchrony and spike-count correlations (r_sc_) are believed to play a role in stimulus selection by increasing the signal and reducing the noise in a population, respectively. Here, we investigate (1) whether feature-based attention biases the correlated activity between neurons when attention is directed towards their common preferred feature, (2) the interplay between spike-synchrony and r_sc_ during feature selection, and (3) whether feature attention effects are common across the visual and tactile systems. Single-unit recordings were made in secondary somatosensory cortex of three non-human primates while animals engaged in tactile feature (orientation and frequency) and visual discrimination tasks. We found that both firing rate and spike-synchrony between neurons with similar feature selectivity were enhanced when attention was directed towards their preferred feature. However, attention effects on spike-synchrony were twice as large as those on firing rate, and had a tighter relationship with behavioral performance. Further, we observed increased r_sc_ when attention was directed towards the visual modality (i.e., away from touch). These data suggest that similar feature selection mechanisms are employed in vision and touch, and that temporal correlation codes such as spike-synchrony play a role in mediating feature selection. We posit that feature-based selection operates by implementing multiple mechanisms that reduce the overall noise levels in the neural population and synchronize activity across subpopulations that encode the relevant features of sensory stimuli.

## Abstract 2

How neurons pay attention Top-down selective attention mediates feature selection by reducing the noise correlations in neural populations and enhancing the synchronized activity across subpopulations that encode the relevant features of sensory stimuli.

## Author Summary

Attention can select stimuli in space based on the stimulus features most relevant for a task. Attention effects have been linked to several important phenomena such as modulations in neuronal spiking rate (i.e., the average number of spikes per unit time) and spike-spike synchrony between neurons. Attention has also been associated with spike count correlations, a measure that is thought to reflect correlated noise in the population of neurons. Here, we studied whether feature-based attention biases the correlated activity between neurons when attention is directed towards their common preferred feature. Simultaneous single-unit recordings were obtained from multiple neurons in secondary somatosensory cortex in non-human primates performing feature-attention tasks. Both firing rate and spike-synchrony were enhanced when attention was directed towards the preferred feature of cells. However, attention effects on spike-synchrony had a tighter relationship with behavior. Further, attention decreased spike-count correlations when it was directed towards the receptive field of cells. Our data indicate that temporal correlation codes play a role in mediating feature selection, and are consistent with a feature-based selection model that operates by reducing the overall noise in a population and synchronizing activity across subpopulations that encode the relevant features of sensory stimuli.

## Introduction

We are constantly exposed to a diverse set of stimuli that excite all of our senses. To effectively function in this environment it is critical that we employ filtering mechanisms such as selective attention to extract the most relevant information to our goals. Specifically, attention has been shown to increase the firing rate (FR) and spike-spike synchrony between cells (e.g., spike-synchrony) and decrease the correlated noise activity between neurons sharing similar somatotopic [1]–[3] or visual-spatial receptive fields (RFs) [4]–[12]. Further, in the visual system, feature-based attention can enhance and suppress neurons' FRs when the focus of attention is directed towards the cells' preferred and non-preferred stimulus feature, respectively [13],[14]. For instance, attention towards a stimulus moving in a particular direction increases the FR of cells that are tuned for stimuli moving in that direction. This mechanism, known as the feature similarity gain model, predicts gain-related attention effects in visual cells [15] and may be a common mechanism across species (see [16],[17] for examples in humans).

A recent study conducted in mouse primary visual cortex (V1) found that orientation selective cells that share similar angle preferences are significantly more interconnected with each other. This study also showed a similar relationship in neurons that displayed analogous responses to different naturalistic stimuli [18]. This connectivity pattern is akin to that of V1 orientation-tuned cells in other mammals [19],[20], where enhanced synchronized spiking activity between neurons tuned for similar orientations was found [19]. Similarly, cells in primary and secondary somatosensory cortex (SII) show tuning for distinct features such as orientation and frequency [21]–[27], and putatively, as in V1, neurons with similar feature selectivity may be preferentially connected. We therefore questioned whether attention takes advantage of such preferential coupling in SII cortex by further modulating the correlated activity between cells tuned for the relevant modality features of a task (e.g., orientation versus vibratory frequency). We hypothesized that spike-synchrony between SII cells selective for the same feature modality would be increased when attention was directed to that modality. Further, based on results in the visual system [9]–[12], we predicted that attention would decrease the spike-count correlation (r_sc_) between neurons across trials (also termed noise correlations). A recent study found reduced r_sc_ between cells with enhanced FRs when attention was directed towards a particular feature of the stimulus [10]. Here, we assessed whether the visual and tactile systems employ analogous mechanisms of feature selection by examining whether similar r_sc_ effects are observed in the somatosensory system, and whether attention effects on FR are predicted by the feature similarity gain model [11],[13],[15].

Another goal was to examine the relationship between spike-synchrony and r_sc_ during attention and their correlation with behavioral performance. Spike-synchrony and r_sc_ measure correlated activity between neural populations but at different temporal scales, with spike-synchrony defined as concomitant spikes within a narrow window (e.g., ±2 ms) and r_sc_ characterized as correlated mean spiking activity across broader timescales (>100 ms). Indeed, studies show that spike-synchrony and r_sc_ can be linearly related [28] and this relationship is enhanced by the tuning similarity between cells [29]. However, these findings seem difficult to reconcile with the observations that attention reduces r_sc_ [9],[12] but also increases spike-synchrony [2]. One possibility is that attention modulates these correlation codes according to the feature selectivity of the population. Indeed, in one study [28] activity was recorded from medio-temporal (MT) neurons with similar RFs that were tuned for the same feature modality (i.e., motion). In contrast, in the visual area 4 (V4) studies [9],[12] it was investigated how attention modulates r_sc_ across cells with the same RF, without regards to their feature modality selectivity. Thus, it is possible that reductions in r_sc_ by attention were predominately between neurons that did not share the same feature selectivity.

Our findings reveal that attention enhances both FR and spike-synchrony when it is focused towards the preferred feature modality of cells. In addition, we found that attention effects in spike-synchrony correlated well with behavior. Consistent with previous reports in vision [9],[12], r_sc_ in SII cells increased when attention was directed towards the visual modality (i.e., away from the somatotopic RF of the neurons). Importantly, these results were observed across animals performing slightly different attention tasks, suggesting that these attention mechanisms are prevalent across perceptual tasks. Taken together, our data are consistent with a feature selection model that operates by reducing the background correlated noise levels in the population and selectively increasing the FR and synchronous activity between cells that encode the stimulus features relevant for the task at hand.

## Results

Three animals (*Macaca mulatta*) were trained to perform tactile and visual discrimination tasks. In the tactile modality animals 1 and 2 performed orientation and/or frequency discrimination tasks, whereas animal 3 performed a match-to-sample (MTS) orientation task. In the visual modality all animals performed a brightness discrimination task.

**Figure 1:**
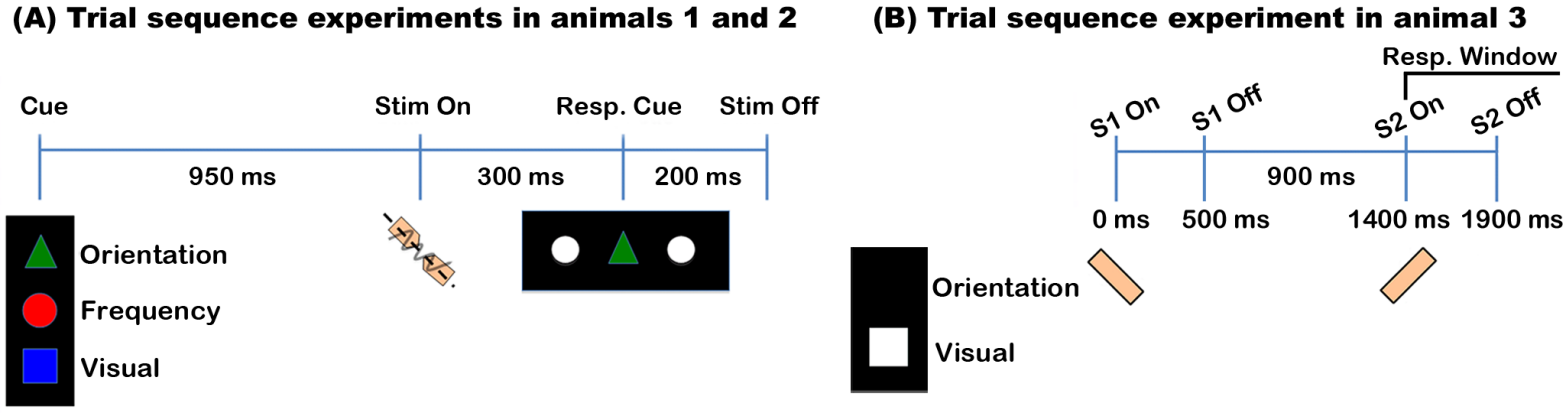
Experimental designs. (A) A trial began with the presentation of a visual cue, in the center of the screen, consisting of one of the three shapes (with different colors) shown. A green triangle instructed the animal to engage in the orientation discrimination task while a red circle indicated the frequency discrimination task. The blue square indicated the visual discrimination task. Following 950 ms the onset of the cue, a vibrating oriented bar was presented for 500 ms to the fingerpad. A visual response cue consisting of two circles flanking the visual cue was presented 300 ms after the onset of the tactile stimulus (500 ms for animal 2), and the animal was required to make a saccade to either of the two flanking circles, depending on the task being performed (see text). The animal was required to main fixation during the entire trial and it was rewarded with a drop of liquid if it responded correctly within 1,000 ms after the onset of the response cue. (B) A trial began with an oriented bar indented on one of the animal's distal fingerpads for 500 ms. After a delay period of 900 ms a second oriented bar was indented on the same fingerpad and with the same duration. The second stimulus had the same or an orthogonal orientation (i.e., difference of 90°) to the first stimulus. The animal displaced a foot pedal in the forward or backward direction if the stimuli had the same or different orientation, respectively. In attend visual trials, the animal experienced the same tactile stimulation, but it was trained to displace the foot pedal when a white square (2° visual angle), which was continuously presented on the screen, was dimmed. A drop of liquid was given for every correct trial.

Figure 1A shows the sequence of events in the experiment performed by animals 1 and 2. A trial began with the presentation of a visual cue on a monitor placed in front of the animal. This cue instructed the monkey to engage in a tactile-orientation (green triangle), tactile-frequency (red circle), or visual-brightness discrimination task (blue square). Following 950 ms the onset of the cue, a vibrating oriented bar was delivered to one of the animal's distal fingerpads for 500 ms. During attend tactile trials, the animal focused its attention on the tactile feature instructed by the cue and made a response by making a saccade towards one of two white circles that flanked the instructional cue. During the attend orientation task, the animal made a saccade to the left circle if the bar was oriented 135° (i.e., 45° counter clockwise relative to the long axis of the finger) and to the right circle if the bar was oriented 45° clockwise. During the attend frequency task a leftward saccade was made if the vibration was low frequency (10 Hz) and a rightward saccade if the vibration was high frequency (40 Hz). These response criteria were counterbalanced across the two animals. During attend visual trials, the animal was trained to ignore the tactile stimulus and make a saccade towards one of the two flanking circles with the highest brightness level. Note that in the attend orientation and attend frequency tasks the flanking circles were of the same brightness.

Figure 1B shows the sequence of events in the experiment performed by animal 3. A trial began with an oriented bar indented on one of the animal's distal fingerpad for 500 ms (0°–157.5°, in steps of 22.5°). After a delay period of 900 ms a second oriented bar was indented on the same fingerpad and with the same duration. The second stimulus had the same or an orthogonal orientation (i.e., difference of 90°) to the first stimulus. The animal displaced a foot pedal in the forward or backward direction if the stimuli had the same or different orientation, respectively. In attend visual trials, the animal experienced the same tactile stimulation, but it was trained to displace the foot pedal when a white square (2° visual angle), which was continuously presented on the screen, was dimmed. A drop of liquid was given for every correct trial. Unlike the other animals, animal 3 performed the tactile and visual trials on separate blocks, and this was cued by changing the pattern on the screen from an illuminated square (visual task) to a blank screen (tactile task).

Single-unit recordings were obtained from the hand region of SII cortex using a custom built multi-electrode array composed of four (animals 1 and 2) or seven channels (animal 3). Each electrode in the arrays could be independently displaced, thus allowing the experimenter to carefully isolate single units on each electrode contact. Animal 1's hit rates were 82%, 82%, and 84% for the attend orientation, attend frequency, and attend visual conditions, respectively. Animal 2's hit rates were 72% and 70% for the attend frequency and attend visual conditions, respectively. Animal's 3 hit rates were 90% and 72% for the attend orientation and attend visual conditions. Animal 2 did not perform the orientation task because it was not able to perform the task above chance levels during the training phase. Animal 3 was never trained to perform a frequency discrimination task (see Materials and Methods).

A cell was categorized as feature selective if its response to the preferred stimulus value (e.g., 60 Hz vibration in the frequency domain), determined during the characterization of neural feature selectivity protocols (see Materials and Methods), was significantly greater than the response to the least-preferred stimulus value (e.g., a 10 Hz vibration) [11]. Neurons selective for both orientation and frequency features were discarded from further analyses. Our goal was to assess whether gain-related attention effects on tactile cells are predicted by the feature similarity gain model. It is unclear what the model's prediction will be for cells selective for both types of features.

A key difference between this study and those that originally described the feature similarity gain model in the visual modality ([13],[15]) is that our experiment required animals to direct attention towards a feature modality of the stimulus (e.g., orientation or frequency), whereas these previous studies trained animals to discriminate stimuli within one single feature modality (e.g., the direction of motion of visual stimuli). Thus, in these previous studies the effects of attention were quantified by comparing the neural responses between two stimulus values from the same feature modality in vision (e.g., 180° motion direction versus 90° motion direction, see [13],[15]), as opposed to two different feature modalities, as in our experiment. Thus, our task is more comparable to that of [11],[14],[16],[30]. Our terminology is one that describes the orientation feature modality composed of a set of oriented stimulus values ranging from 0° to 180°. In the same spirit, the frequency feature modality is composed of a set of vibrating stimuli ranging from 10 to 100 Hz.

### Feature Selection Effects on the Firing Rate of Somatosensory Neurons

**Figure 2:**
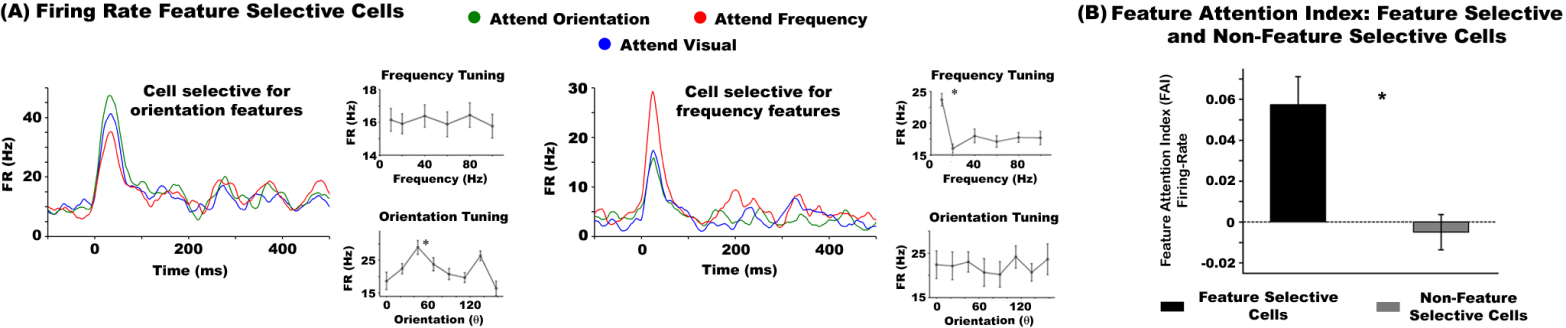
Attention modulates the firing rates of somatosensory cells in a feature-specific manner. (A) The FR profile of two example neurons selective for orientation (left graph) and frequency (right graph) features. Attention to orientation, frequency, and vision are represented in the green, red, and blue traces, respectively. Graphs are aligned to the onset of the tactile stimulus (t = 0). The lower four graphs show the orientation and frequency tuning curves of each cell. These example neurons are from animal 1. See Figure S2 for other example neurons in animals 2 and 3. The instantaneous FR waveforms were smoothed with a ±5 ms moving average filter. (B) FAI of feature selective and non-feature selective neurons. The asterisks denote statistical significance (*p*<0.05). The underlying data used to make this figure can be found in Data S1.

We analyzed the effects of attention on the FR of SII neurons. Figure 2A shows attention effects in two feature selective neurons. The two graphs to the right of each colored graph illustrate the frequency and orientation tuning curves for each neuron. The left graph illustrates a neuron selective for orientation with a preferred angle of 45° (as shown in its orientation tuning graph) and enhanced FR when attention was directed towards orientation compared to frequency. The right graph shows the opposite pattern for a neuron selective for frequency with preferred vibration at 10 Hz (as shown in its frequency tuning graph). The results for the three animals were highly similar (see Figures S2 and S3 and text below), therefore we combined their data for population analyses. The population statistics showed that 43% of all feature selective neurons (*n* = 94) were modulated by attention, with 75% of these neurons having greater FR when attention was directed towards versus away from the cell's preferred feature. Animal 2 received the same pattern of tactile stimulation as animal 1, but during recordings it was never cued to perform the orientation task. Animal 3 performed a match-to-sample tactile orientation task only. In animal 2 we analyzed attention effects by comparing the activity of attend frequency versus attend visual, while in animal 3 we analyzed attention effects by comparing activity between attend orientation versus attend visual. This analysis was largely performed in feature selective cells (i.e., orientation or frequency). However, we performed additional analyses to further assess the validity of these attention effects. In particular, we reasoned that if FR attention effects are indeed feature-specific, animal 2's orientation selective cells should exhibit reduced attention effects, as compared to frequency selective neurons, when attention was apportioned to frequency versus vision. We observed that only 22% (two out of nine cells) of animal 2's orientation selective cells had significantly greater FRs when attending towards frequency versus vision. In contrast, 38% of frequency selective cells in animal 2 had increased FRs when attention was deployed to frequency versus vision (five out of 13 cells). Animal 1 displayed similar attention modulations in its feature modality selective cells, with 16% of orientation selective (three out of 19) and 38% of frequency selective cells (six out of 16) displaying higher FRs when attention was deployed to frequency versus vision.

Similar to animal 2's hypothesis, we reasoned that non-feature selective cells in animal 3 should exhibit enhanced activity, or a null effect, when attention was directed to vision as compared to orientation. Consistent with this hypothesis, 54% of non-orientation selective cells in this animal did not exhibit attention effects, while 28% had significantly greater activity when attention was apportioned to vision as compared to orientation.

The magnitude of attention effects was quantified using a feature attention index (FAI) [31]. This was calculated by subtracting the mean response when attention was directed away from a neuron's preferred feature modality from the mean response when attention was directed towards the preferred feature modality, and dividing the difference by the sum of these two quantities. In animal 2 the FAI was calculated by subtracting the mean response to attention towards vision from attention to frequency and dividing the difference by the sum of these quantities. In animal 3 the FAI was calculated using the same formula but substituting attention to frequency with attention to orientation. Further, because by default, non-feature selective cells do not have a preference for orientation or frequency stimuli, we devised a surrogate “preferred feature modality” to calculate the FAI in these cells. We reasoned that classifying the “preferred feature modality” of non-feature selective cells in this way would lead to a consistent pattern of feature-based attention effects that would be comparable to those observed in feature selective populations.

The “preferred feature modality” in non-feature selective cells was assigned by first computing a feature modality selectivity index (FMSI) value for both orientation and frequency conditions, and then labeling the condition with highest FMSI as the “preferred feature modality.” The FMSI was computed by subtracting the response to the stimulus that evoked the weakest activity (e.g., a 60 Hz vibration in the case of frequency; a 22.5° oriented stimulus in the case of orientation) from the stimulus that elicited the strongest response within the same feature modality (e.g., 40 Hz vibration in frequency, or 90° in orientation) and dividing this difference by the sum of the two quantities. This analysis was done from data collected in the feature selectivity characterization protocols. To derive the FAI for a non-feature selective neuron we subtracted the response to attention towards the “least preferred feature modality” (i.e., the feature with lower FMSI value) from attention towards the “preferred feature modality” (i.e., the feature modality with higher FMSI value) and dividing the difference by the sum of the two quantities. Unfortunately, this FMSI analysis could not be performed in animal 3 because the frequency selectivity protocol was not performed in this animal. In this animal we assessed FAI in non-feature selective neurons by subtracting the mean response when attention was directed towards orientation from the mean response when attention was directed towards the vision, and dividing the difference by the sum of these two quantities.

The mean FAIs for feature selective and non-feature selective populations were 0.057 and −0.009, respectively. The FR FAI values were not normally distributed, thus we conducted a Mann-Whitney U-test to test for significant differences in the effects of attention between the two cell populations. The analysis revealed a significant difference, whereby feature selective cells exhibited higher FAI (Z = 3.42, *p* = 0.0006; see Figure 2B). The effect size, measured as Cohen's d, was 0.59.

We assessed whether there was a relationship between a cells' FMSI and its FAI. To do this we sorted FAIs as a function of the difference between the highest and lowest FMSI (i.e., the preferred and non-preferred FMSI). This analysis was performed in both feature and non-feature selective populations and in animals 1 and 2 only. As described above, an FMSI could not be computed for animal 3. Linear regressions, using FAI as the response variable did not reveal a systematic relationship in feature selective (R^2^ = 0.02, *p*>0.32) or non-feature selective populations (R^2^ = 0.001, *p* = 0.64). These data are illustrated in the left panel of Figure S4.

**Figure 3:**
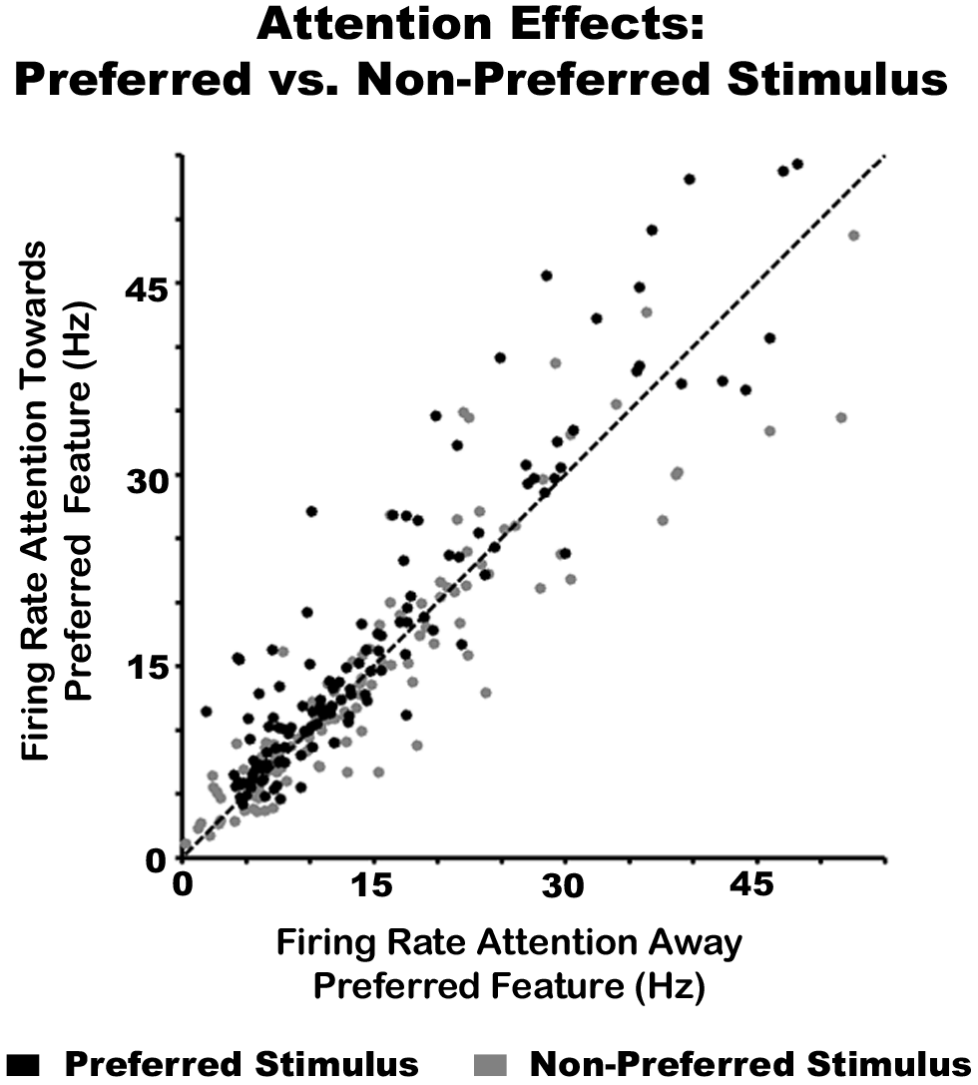
Attention effects are greater on the preferred stimulus value. The black and gray dots represent the FR of a neuron to its preferred and non-preferred stimulus value, respectively. The x-axis shows the response when attention was deployed away from the preferred feature modality, while the y-axis represents the response when attention was biased towards the preferred feature modality of the neuron. The dotted line is the identity. The underlying data used to make this figure can be found in Data S1.

We further tested whether attention effects in the FRs were stimulus-value specific. For this analysis we identified neurons whose greatest response to a stimulus value during the feature selectivity characterization protocols was tested during the attention experiment (e.g., 45° or 90° in an orientation selective neuron, number of cells = 130), regardless of whether they were classified as feature selective by our definition. The FR response to the non-preferred stimulus (e.g., 135°) was subtracted from that of the preferred stimulus (e.g., 45°) and we conducted a Mann-Whitney U-test between Attention towards the preferred versus non-preferred feature modality conditions. The data revealed significantly greater attention effects on the preferred stimulus versus non-preferred stimulus value (Z = 4.93, *p*<0.0001). Figure 3 shows the FRs for the preferred (black dots) and non-preferred (gray dots) stimulus value when the animal attended towards the preferred feature versus away from the preferred feature modality. The figure shows the black dots consistently above the unity line. These findings agree with those reported by [13] in the visual system, where FR attention effects on MT neurons were found to be stimulus-value specific. Taken together, these results provide evidence that the feature similarity gain model also operates in the somatosensory system, suggesting that both vision and touch employ similar gain-related mechanisms of feature selection.

### Feature Selection Effects on Spike-Synchrony

Spike-synchrony was defined as the number of times two neurons fired an action potential (AP) within ±2 ms of each other on a 1 ms sliding time scale for every trial and averaged over all trials. This technique has the advantage over other methods (e.g., cross-correlograms [CCGs], which averages over all time scales) in that it maintains the temporal structure of spike-synchrony events, thus allowing us to assess changes in synchrony across time, instead of the mean coincident spikes across the entire spike train. Spike-synchrony due to “chance” for each attention condition was estimated using the jitter-correction method [32], and this “chance” synchrony was subtracted from the observed spike-synchrony. Only neural pairs whose jitter-corrected spike-synchrony was significantly greater than zero for at least 100 ms (*p*-value level of 0.05) in at least one attention condition were analyzed for attention effects on spike-synchrony (*n* = 47 out of 57 feature selective neural pairs, and *n* = 57 out of 65 non-feature selective neural pairs). We classified a feature selective neural pair as one in which two simultaneously recorded neurons had selectivity for the same feature modality (e.g., orientation). In contrast, a neural pair in which two neurons were not selective for both frequency and orientation tactile features was categorized as non-feature selective. These include neural pairs in which one neuron was selective for a particular tactile feature but the other cell was not.

**Figure 4:**
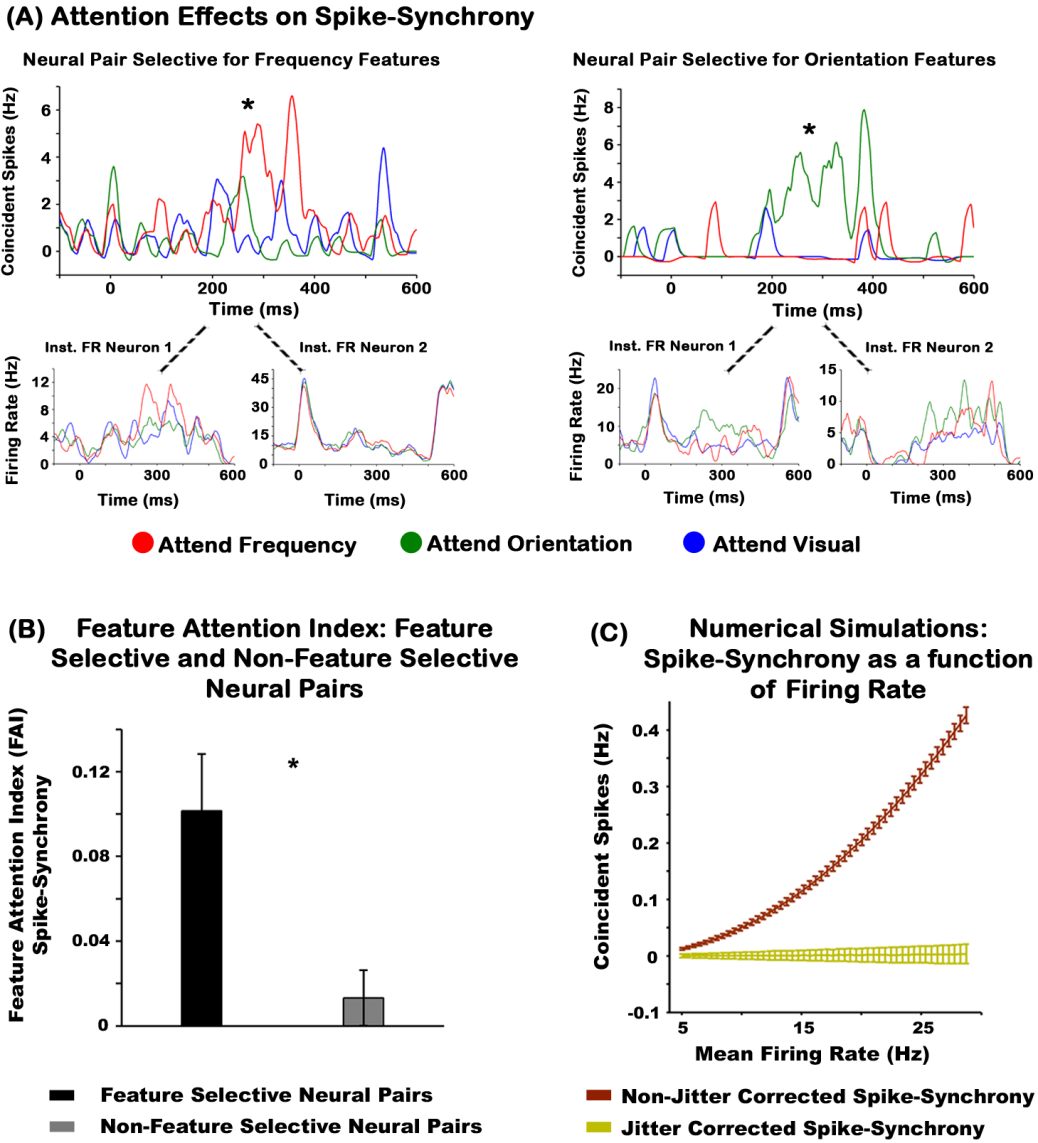
Attention modulates spike-synchrony rates of feature selective neural pairs. (A) Instantaneous spike-synchrony activity of two neural pairs selective for frequency (left graph) and orientation (right graph) features. Attention towards orientation, frequency, and visual stimuli is represented in green, red, and blue traces, respectively. The “chance” synchrony for each attention condition was subtracted. The lower panels show the instantaneous FR profiles of each neuron comprising the neural pair. All graphs are aligned to the onset of the tactile stimulus (t = 0). These example neural pairs are from animal 1. See Figure S3 for other example neural pairs in animals 1, 2, and 3. The instantaneous FR and synchrony waveforms were smoothed with a ±5 ms moving average filter. (B) FAI of feature selective and non-feature selective neurons. (C) Numerical simulations of spike-synchrony between two neurons as a function of the averaged FR between the neural pair. The graphs show that the jitter correction method [32] removes the spike-synchrony expected by chance across all FR values. The asterisks in Figure 4A indicate significant differences between spike-synchrony across the attention conditions (*p*<0.05). The asterisk in (B) indicates a significant difference in the FAI between the two neural populations. The underlying data used to make this figure can be found in Data S1.

Similar to the FR results, we observed feature specific attention effects in spike-synchrony. Figure 4A illustrates the instantaneous spike synchrony of two feature selective neural pairs for all attention conditions. The left graph shows that attention towards frequency evoked the largest spike-synchrony for a neural pair selective for frequency. The right graph shows that attention towards oriented features yielded the highest spike-synchrony for a neural pair selective for orientation. The lower panels of Figure 4A show the instantaneous FR profiles of each neuron comprising the neural pair. While increases in FR and spike-synchrony often occurred around the same time, jitter correction methods applied to the synchrony data [32] show that attention effects on synchrony are not explained by FR modulations alone (see below). We found that the mean spike-synchrony rates of feature selective neurons were 5.51 times greater than the spike-synchrony due to “chance” computed from the jitter method [32]. Figure S3 shows other example neural pairs from both animals illustrating similar feature selective effects. The population data revealed attention effects on spike-synchrony in 55% of all feature selective neural pairs (26 out of 47). Of these, 77% had greater synchrony rates when attending towards versus away from the preferred feature modality, and a Pearson's chi-squared test revealed that this difference was significant (χ^2^ = 7.53, *p* = 0.003). The degree of synchronous firing was not correlated with the anatomical distance between neural pairs (Figure S5).

Attention effects on spike-synchrony were quantified using the same FAI formula for the FR data. This is illustrated in Figure 4B for feature selective and non-feature selective neural pairs. The average FAI of feature selective populations was 0.102, whereas the mean FAI for non-feature selective cells was 0.013. Similar to attention effects on FR, we observed that FAI values for spike-synchrony were not normally distributed. A Mann-Whitney U-test revealed greater FAI for feature selective neural pairs as compared to non-feature selective cells (Z = 2.06, *p* = 0.039), with an effect size of 0.58 as measured by Cohen's *d*.

We further tested whether there was a relationship between FMSI and spike-synchrony FAI. We calculated the average of the two neurons' FMSI for each feature condition, and sorted the FAI as a function of the difference between the preferred and non-preferred FMSI. This analysis was performed in feature selective and non-feature selective neurons. As described above, this analysis was only performed in animals 1 and 2 because an FMSI could not be computed for animal 3. Linear regressions did not reveal a significant relationship in feature selective (R^2^ = 0.032, *p* = 0.28) or non-feature selective cells (R^2^ = 0, *p*>0.95). These data are illustrated in the middle panel of Figure S4.

Computational and experimental studies have shown that increases in spike-synchrony can be caused by increases in neurons FR and/or slow co-variation artifacts [33]–[35]. To test against these confounds, we corrected our spike-synchrony data by employing the temporal jitter method developed by Amarasingham and colleagues [32], which removes the effects of slow wave co-variations beyond the correlation window chosen by the experimenter (in our case 50 ms). We performed a series of numerical simulations to show this method is also robust against enhancements in neurons' FR (see Figure 4C). In these simulations, two independent spike trains were generated using a non-homogeneous Poisson process (250 trials), which simulated the FR profile of a neural pair. A non-homogenous Poisson rate function was used to have a better approximation of the spiking behavior of a typical neuron. The FR of each spike train was systematically modulated from 5 to 28.65 Hz. For each of the 250 trials the spike-synchrony between the two spike trains was calculated using a ±2 ms bin window (the same used in the analyses of our experimental data), and then averaged across all trials. These procedures were repeated 5,000 times and averaged. As expected, spike-synchrony increased as a function of FR (Figure 4C, brown trace). However, the jitter correction method removed this dependence (mustard color trace). Taken together these findings indicate that our spike-synchrony results are not accounted for by increases in FR or, as shown by [32], slowly co-varying changes in FR.

### Feature Selection Effects on Spike-Count Correlations

**Figure 5:**
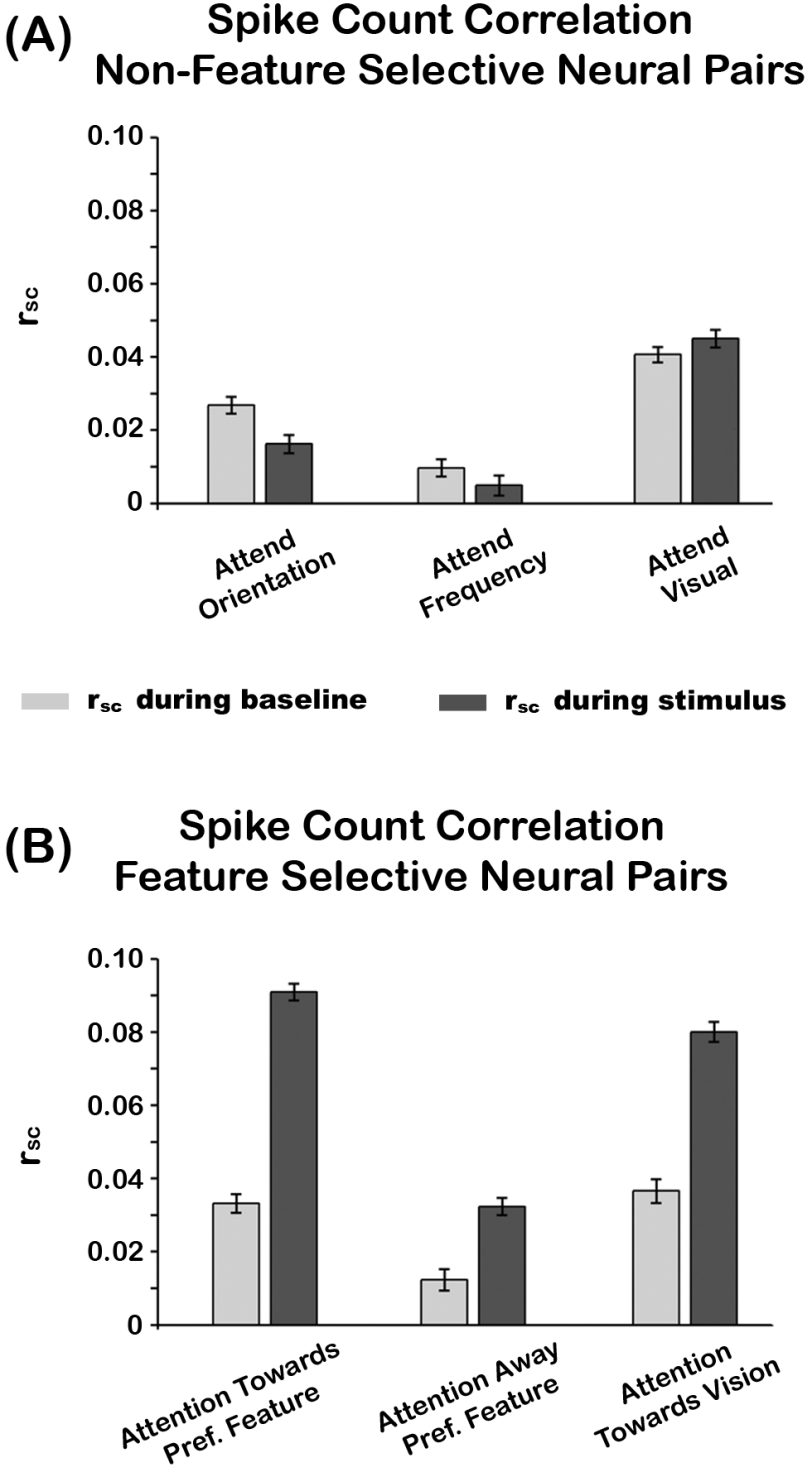
Attention effects on spike-count correlations. (A) Attention effects on r_sc_ of non-feature selective neural pairs. Light and dark gray bars are r_sc_ during baseline and stimulus presentation period, respectively. (B) Attention effects on r_sc_ of feature selective neural pairs. Same color convention as above. In all graphs, error bars represent the within subject standard error of the mean (SEM_within_). The underlying data used to make this figure can be found in Data S1.

r_scs_ were computed in feature selective and non-feature selective neural pairs during the stimulus presentation and baseline period (using the averaged activity from −500 to 0 ms prior to visual cue onset). We observed that r_sc_ values were normally distributed for both non-feature selective and feature selective populations. A two-way repeated measures ANOVA with factors of attention (orientation, frequency, and visual) and time (baseline versus stimulus presentation period) on non-feature selective populations did not reveal a significant main or interaction effect for any condition (see Figure 5A). In contrast, a two-way repeated measures ANOVA with factors of attention (attention towards the preferred feature modality, attention away from the preferred feature modality, and attention to vision) and time (baseline versus stimulus presentation period) on feature selective populations revealed a main effect of attention (F(2,112) = 3.59, *p* = 0.031) and a main effect of time (F(1,56) = 5.62, *p* = 0.021) (see Figure 5B). Post hoc paired sample t-tests revealed that the main effect of attention was driven by higher r_sc_ in the attend towards versus attend away from the preferred feature condition (t(56) = 2.46, *p* = 0.02), as well as higher r_sc_ in the attend visual versus attend away from the preferred feature condition (t(56) = 2.23, *p* = 0.030). The main effect of time was driven by higher r_sc_ during the stimulus presentation period (t(56) = 2.37, *p* = 0.021). No other significant effects were observed.

We also assessed whether there was a relationship between FMSI and attention effects on r_sc_. We performed the same analyses as in the spike-synchrony data. Linear regressions failed to reveal a relationship between attention effects on r_sc_ and FMSI in feature selective (R^2^ = 0.02, *p* = 0.39) and non-feature selective neural pairs (R^2^ = 0.02, *p* = 0.75). These data are illustrated in the right panel of Figure S4.

### Relation between Spike-Synchrony and Spike-Count Correlation

**Figure 6:**
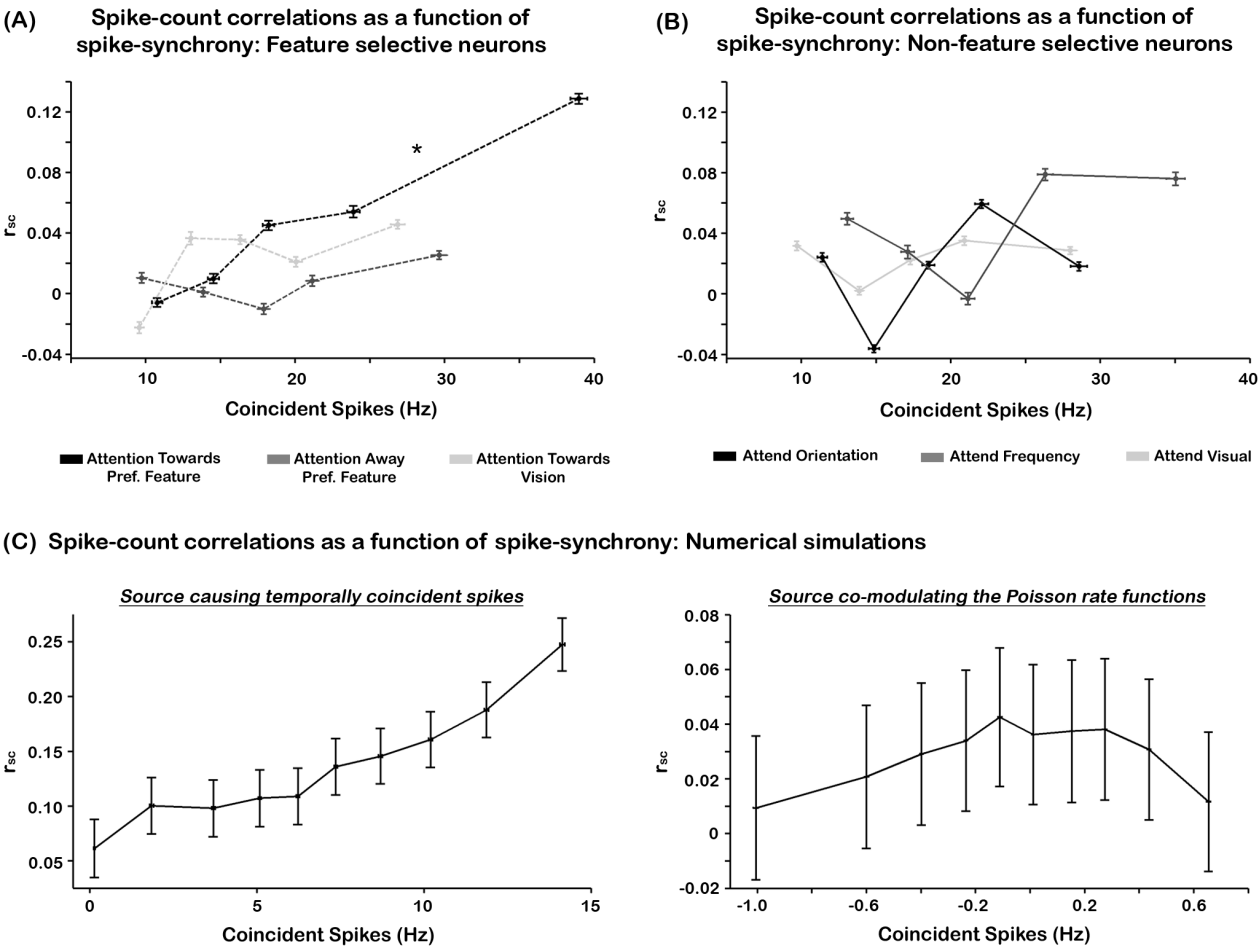
Relation between spike-synchrony and spike-count correlations. (A) r_sc_ as a function of spike-synchrony in feature selective populations. The individual traces represent the binned r_sc_ for the “attention towards preferred feature” (solid black trace), “attention away preferred feature” (dashed dark-gray trace), and “attention towards vision” (dashed light-gray trace). The results show that greater spike-synchrony led to enhanced r_sc_ values only when attention was directed towards the preferred feature modality of the neural pairs (solid black trace). This is indicated by the asterisk (*p*<0.05). (B) r_sc_ as a function of spike-synchrony in non-feature selective populations. Attention to orientation, frequency, and vision are represented in black, light gray, and dark gray, respectively. The horizontal and vertical error bars in Figure 6A and 6B represent the SEM_within_. (C) Numerical simulations of r_sc_ as a function of spike-synchrony. The graphs show two scenarios that either caused temporally coincident spikes across two AP trains (left panel) or co-modulated the FRs of two spike trains (right panel). The simulations revealed a systematic relationship between r_sc_ and spike-synchrony in the first scenario only. The error bars in all graphs represent 95% confidence intervals. The underlying data used to make this figure can be found in Data S1.

The effects of attention on r_sc_ in feature selective neural pairs are not in entire agreement with the findings presented in [11]. Briefly, that study found decreased r_sc_ in neural pairs that displayed concomitant increases in FR when attention was apportioned to a particular feature of a visual stimulus. We reasoned that because spike-synchrony is in itself a correlation mechanism, but at a faster timescale, enhancements in r_sc_ by attention might reflect the increases in synchrony rates observed in the same neural population. To test this hypothesis we assessed whether increases in spike-synchrony were temporally correlated with enhancements in r_sc_. We computed the r_sc_ and spike-synchrony rates every 100 ms during the stimulus presentation period in every feature selective neural pair. Then, we sorted r_sc_ values as a function of the time-binned spike-synchrony rates and averaged these values across neurons. These data, illustrated in Figure 6A, show a positive relationship between jitter-corrected spike-synchrony and r_sc_ but only when attention was directed to the preferred feature modality of the neural pair. A regression analysis showed that this relationship was well-fitted by a linear function (F(5, 41) = 8.58, *p*<0.001, R^2^ = 0.51). These results suggest that increases in spike-synchrony underlie the enhancements in r_sc_ but only when attention is directed toward the preferred feature of cells.

We tested whether the relationship between spike-synchrony and r_sc_ was exclusive to feature selective neurons by computing the same analysis as above in non-feature selective neural pairs. Regression analyses did not reveal a statistical effect in any attention condition (*p*>0.05; R^2^ = 0.09, 0.21, R^2^ = 0.17 for attend-orientation, frequency, and visual, respectively, see Figure 6B).

We performed a series of numerical simulations to determine possible neural mechanisms that may account for the correlation between spike-synchrony and r_sc_. We implemented scenarios where correlated spiking activity within a neural population was produced by either a source that (1) caused a coincident volley of spikes across the population or (2) co-modulated the mean FR function of all neurons, resulting in a correlated change in the probability of generating coincident spikes across the population. The former is comparable to a neural population receiving strong common monosynaptic inputs, whereby cells' membrane potentials are raised above depolarization threshold level around the same time (i.e., a supra-threshold influence). In contrast, the latter is akin to a probabilistic model that modulates the membrane potentials of cells without necessarily causing cell depolarization. This latter model is similar to those implemented in previous studies [12],[33]. A visual illustration of the two scenarios is shown in Figure S6. For both scenarios, we constructed two independent spike trains (250 trials) generated by a non-homogenous Poisson process with a mean FR of 25.58 Hz, constructed to mimic the FR profile of a typical neuron responding to a stimulus. This FR profile and an example raster plot are shown in Figure S6A. In the first scenario the source was a binary waveform whose value was usually zero, but periodically jumped to one and added 0 to 10 spikes (uniformly distributed) in both spike trains every 400 ms (i.e., 2.5 Hz). In the second scenario the rates of both spike trains were multiplied by a 2.5 Hz sine wave with amplitude ranging from 0.5 to 1. We chose a 2.5 Hz signal based on the findings by [12], which showed that most of the correlated spiking activity across a neural population is captured in the ongoing oscillating activity between 0 and 5 Hz. Similar to the analysis performed on our experimental data, we sorted the r_sc_ as a function of the jitter-corrected spike-synchrony and divided the data across ten bins. These procedures were repeated 5,000 times. The results from scenario 1 revealed a systematic linear relation analogous to that observed in our experimental dataset (R^2^ = 0.91, F(1,8) = 89.75, *p*<0.001) (Figure 6C, left graph). However, the results from scenario 2 did not reveal any systematic pattern between r_sc_ and spike-synchrony (R^2^ = 0.09, F(1,8) = 0.76, *p*>0.05) (Figure 6C, right graph). In fact, these data show a very narrow window of modulations in spike-synchrony (−1 to 0.7 Hz; note differences in scales on both axes between left and right panel), which further supports the use of the jitter correction method for removing spurious spike-synchrony activity due to slow co-variation signals. Taken together, these data indicate that attention effects on the spike-synchrony and r_sc_ observed in feature selective neurons might be mediated by an external neural population that induced coincident spikes across the feature selective neural set.

### Relation between Neurophysiology and Behavior at the Single-Trial Level

Finally, we examined the relation between FR and spike-synchrony, both of which showed feature selective attention effects in the expected direction, and behavior. Correct and incorrect trials were sorted as a function of FR and jitter-corrected spike-synchrony separately (see [36],[37] for a similar analysis). The sorted data were divided into five bins of equal sizes to reduce the effects of outliers, and the percentage of correct trials within each bin was calculated. This procedure was performed in feature selective cells with at least 25 trials per attention condition. This resulted in 86 single neurons and 42 neural pairs for the FR and spike-synchrony analyses, respectively. Regression analyses were performed using percent correct as the response-variable and the neurophysiology as predictor (FR or synchrony rate). The regression analyses on the FR data revealed a significant relationship with behavior when attention was directed to the preferred feature of cells (F(5,80) = 4.19, *p*<0.05, R^2^ = 0.20). In addition, we observed an inverse relationship between FRs and behavior when attention was directed to vision (F(5,80) = 4.11, *p*<0.05, R^2^ = 0.20). However, the range between the lower and higher behavior bins for both attention conditions were ∼3%, indicating that FR attention effects had a very narrow window for modulating behavior.

**Figure 7:**
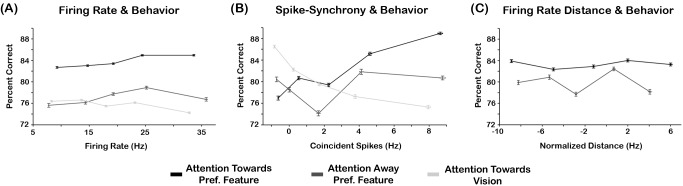
Relation between neurophysiology and behavior. (A) Percent correct as a function of the mean FR between neural pairs. The individual traces represent the percent correct for the “attention towards the preferred feature” (solid black trace), “attention away from the preferred feature” (dashed dark-gray trace), and “attention towards vision” (dashed light-gray trace). (B) Percent correct as a function of spike-synchrony. The individual traces are illustrated in the same convention as above. (C) Percent correct as a function of normalized FR distance. The individual traces are illustrated in the same convention as above. We employed “attend towards vision” as a reference for the proximity values of attention “towards” and “away” from the preferred feature. Thus, the relationship between behavior and distance for “attend towards vision” was not computed. The horizontal and vertical error bars represent the SEM_within_. All data were derived from feature selective neural pairs only. The underlying data used to make this figure can be found in Data S1.

The regression analyses on the spike-synchrony data showed a positive relationship for the attend towards the preferred feature modality (F(5,37) = 3.17, *p*<0.05; R^2^ = 0.29) and an inverse relationship for the attend towards vision conditions (F(5,37) = 3.28, *p*<0.05; R^2^ = 0.30; see Figure 7B). However, the range between the lower and higher behavior bins for both attention conditions was almost four times the range of modulation in the FR. No other significant relationships were observed. Note that negative spike-synchrony values indicate that the average synchrony in those bins was lower than the jitter-corrected spike-synchrony.

To study the links between r_sc_ and behavior we implemented a design similar to that in [11]. Briefly, for each trial we computed the FR response of a neural pair and projected that value to an “attention” axis, which was derived by drawing a line that linked the mean FR response of all correct trials of the attend visual and the attend tactile (i.e., orientation or frequency, separately). The FR of a single trial in the attend orientation or attend frequency was assigned a proximity value, which was the distance from its location on the attention axis to the mean of the attend visual and attend tactile response. If the point was closer to the mean of the attend vision condition then it was assigned a negative value. If it was closer to the attend tactile response then it was given a positive value. The behavior for each trial was then sorted with respect to the distance values, and averaged across five bins of equal size. These data were submitted to a regression analysis using distance values as the predictors and the behavior as the response variable. Only feature selective neural pairs with at least 25 trials per condition were included in the analysis. This resulted in 42 neural pairs. As shown in Figure 7C the proximity analyses failed to reveal a significant relationship with behavior for any attention condition (*p*>0.05). Note that we used attend to vision as a reference for the proximity values, thus the behavior/distance relationship for this condition was not computed.

## Discussion

We studied mechanisms of feature selection in somatosensory cortex in animals trained on several feature discrimination tasks. To the best of our knowledge, no studies have attempted to simultaneously record from multiple well-isolated single neurons, characterize their selectivity to two sensory features, and examine their activity while attention is biased towards or away the common preferred feature of the neural pair. The data showed that FR and spike-synchrony were enhanced when attention matched the preferred feature modality of cells. However, attention effects on spike-synchrony were twice as large, and had a larger window for modulating behavior. Further, in support of previous studies [11],[12], r_sc_ between somatosensory cells was increased when attention was directed away from the RF of neurons (by directing attention towards the visual modality).

### Gain-Related Feature-Based Attention Mechanisms Share Commonalities across Sensory Systems

Our data suggest that gain-related feature selection mechanisms are analogous across the visual and somatosensory systems. Similar to previous findings in visual cortex [11],[13],[15], we observed that attention modulated a large set of neurons in the tactile modality according to the feature similarity gain model, whereby higher FRs were elicited when attention matched a neuron's preferred feature modality. Further, we found that attention effects in somatosensory cortex are stimulus specific with greater modulations on the preferred versus non-preferred stimulus value (a measure of attention effects within a feature modality). However, we found that these feature-specific attention effects did not scale with the FMSI of neurons (a measure of attention effects between feature modalities). This finding suggests that attention modulation is more pronounced within versus between feature modalities (e.g., 45° versus 135° in the orientation modality as compared to orientation versus frequency modalities). Taken together, our results indicate that attention biases the activity of the entire set of neurons selective for features in the attended modality, but these effects may be further enhanced within the sub-population encoding the relevant stimulus values of the task.

That both tactile and visual sensory systems are governed by similar feature-based attention mechanisms promotes the hypothesis that feature selection is controlled by a common set of feature-specific neural areas, whose neurons encode similar stimulus features across the senses (e.g., oriented stimuli in vision and touch). The neural areas containing these putative cross-modal feature selective neurons are unknown, but the lateral prefrontal and lateral intra-parietal cortices are likely candidates since they engage in top-down attention and encode inputs from multiple sensory modalities [38]. Indeed, a recent single-unit study in non-human primates found that neurons in the prefrontal cortex encode information about the relevant stimulus in a visual discrimination task [30]. A question that merits further investigation is whether the tactile and visual systems employ similar neural mechanisms mediating other forms of stimulus selection such as spatial, temporal, and object-based attention.

### Spike-Synchrony Mechanisms and Their Role in Feature Selection

Our dataset showed that attention modulated spike-synchrony in a feature-specific manner, whereby higher synchrony was observed when attention matched the preferred feature modality of the neural pairs. The data showed that the magnitude of this attention effect was predictive of animals' behavior, with greater spike-synchrony associated with improved performance. Equally important, the opposite relationship was observed when attention was directed towards vision. These findings highlight the effectiveness of the attention system to enhance the neural circuits engaged in processing relevant stimuli associated with the task goals but also to suppress unrelated or distracting inputs.

An important observation is that the behavioral performance of animals was not strictly contingent on the amount of spike-synchrony in the population. As Figure 7B shows, even in the absence of spike-synchronous events (see e.g., the first bin that shows spike-synchrony below chance levels), the behavioral performance was well-above chance, indicating that additional mechanisms may mediate behavior (possibly those mechanisms reducing r_sc_ as previous studies show [11]). An alternate explanation is that we only record from a subset of all neurons in various areas of the brain that lead to the animal's behavior. It would be interesting to assess, as Cohen and Maunsell found for r_sc_ [11], whether spike-synchrony attention effects and their relationship to behavior are better accounted for by increasing the pool of neurons exhibiting synchronous spikes. Unfortunately, we are not able to answer this question because our experimental setup very rarely allowed us to record activity from more than two neurons at the same time.

### The Role of Spike-Count Correlations in Feature Selection

Attention has been shown to decrease r_sc_ when it is apportioned to the relevant spatial location of visual stimuli [39]. Our data partially support these findings by showing that r_sc_ between feature selective SII cells was increased when animals performed the visual task. Unexpectedly, our data also revealed increased r_sc_ when attention was directed towards the preferred feature of neural pairs. This finding is inconsistent with results in V4 reported in [11], which found reductions in r_sc_ in neurons that displayed feature-specific FR effects. Specifically, the authors reported an inverse relationship between attention effects on r_sc_ and FR, with greater FR attention modulations associated with lower r_sc_. This pattern of effects led the authors to conclude that attention decreases r_sc_ in neural pairs whose tuning matches the attended feature, a conclusion that does not align with our findings. A putative factor underlying differences between the studies may be in the definition of feature selective neurons. Cohen and Maunsell [11] determined feature preference based on the effect that attention had on their FR, whereby greater FR attention effects to a particular feature (e.g., orientation) implied neural selectivity for that feature. We, on the other hand, defined neural feature selectivity, independently of attention, based on the neuron's responses to a collection of orientation and frequency stimuli presented during sessions where animals were not performing a tactile attention task. Another possibility leading to differences is that we only analyzed feature attention effects on fully isolated and well-characterized single-units whereas Cohen and Maunsell [11] analyzed these effects in the pooled activity of both single and multiunit activity.

The increase in r_sc_ when attention matched the preferred feature modality of neural pairs motivates the following question: if r_sc_ reflect “noisy” and redundant information, then why would attention increase the noise across neurons that encode relevant features of stimuli? We surmised that because spike-synchrony is also a correlation mechanism, the enhanced synchrony rates observed in the same neural population might underlie the increases in r_sc_. Experimental observations and simulation analyses provide evidence for this hypothesis (see Figures 6A and 6C). The data showed that increases in r_sc_ coincided with enhancements in spike-synchrony when attention matched the feature preference of cells. These results are in agreement with those by Bair and colleagues [28] who showed a similar linear relationship in MT cells, in non-human primates engaged in a motion discrimination task.

The question emerges then, what neural mechanism(s) gave rise to the pattern of attention effects in spike-synchrony and r_sc_? A putative hypothesis, which is supported by our numerical simulations, is that these effects were driven by a neural population, likely residing in higher-order cortical areas that caused transient but temporally coincident spikes across feature selective cells in SII cortex. Indeed, the addition of common spikes to a population would result in enhanced spike-synchrony because these induced APs would occur almost at the same time across the entire neural cohort. But, in addition, these common spikes would produce increases in r_sc_ because the amount of added APs would co-vary across the population on a trial-by-trial basis. We note that this hypothesis is speculative, and although our simulations provide support for it, more rigorous physiological studies are needed.

It is important to note that we are not claiming that spike-synchrony gives rise to correlated noise activity (i.e., r_sc_). Rather, our explanation is that because spike-synchrony and r_sc_ measure correlated spiking activity using similar mathematical operations (see equations in Materials and Methods section), enhancements in spike-synchrony will lead to increases in r_sc_. However, the opposite is not always the case. That is, enhancements in r_sc_ can occur in the absence of spike-synchrony. This is highlighted in our dataset, which revealed increased r_sc_ without enhancements in spike synchrony during attend visual trials (Figure 5B), and it is also observed in Cohen and Maunsell [11]. This pattern argues in favor of spike-synchrony and r_sc_ being independent temporal correlation mechanisms. However, a key element in this proposal is that the correlated spikes reflecting r_sc_ must occur during time windows wider than those defined for spike-synchrony (i.e., >±2 ms). That is, the correlated spikes within a trial must occur asynchronously. As suggested by the study of Cohen and Maunsell [9], alpha-band (8–14 Hz) oscillations related to sensory suppression may have caused the reductions in r_sc_ observed in our and their studies. This neural mechanism is thought to index suppression of activity in neurons encoding distracting inputs over relatively broad timescales [16],[36],[37],[40]–[43].

In summary, our findings show that attention only decreased r_sc_ when it was deployed away from vision. That is, it did not decrease r_sc_ in a feature-specific manner. However, this does not imply that r_sc_ are inconsequential for facilitating feature selection. In fact, studies by Cohen and colleagues elegantly show the opposite [9],[10],[11],[44]. These studies report strong links between reductions in r_sc_ and behavioral performance at the single trial level. One reason for our failure to reveal a link between r_sc_ and behavior may be due to limitations in our experimental setup to simultaneously record large samples of neurons. As reported in [11], the ability to predict behavior based on r_sc_ depends on the number of neurons used in the analyses. These authors showed that simulations with <∼five neurons yields very poor predictions of behavior (∼50%), but this ability substantially increases with larger sample populations, with an asymptote at ∼80 neurons. Unfortunately, the vast majority of time our recording paradigm only allowed us to record from two cells at a time.

### A Model of Attention Based on Neural Correlation Codes

Studies in the visual system, as well as our own dataset, indicate that feature-based attention operates by increasing the FR responses of neurons when attention matches their preferred stimulus feature [13],[14]. This mechanism is known as the feature similarity gain model of attention. However, while this model is a reliable predictor of gain-related attention effects in single cells, there are considerable disadvantages in employing attention mechanisms solely based on gain modulations. Because mean-rate codes encode the physical attributes of sensory stimuli (e.g., intensity or brightness) and neurons FRs are also modulated by attention, together they produce non-unique solutions to different combinations of stimulus features and attention conditions. This is illustrated in the following example: if a neural population's FR co-varies with both stimulus intensity (e.g., contrast, sound amplitude, or skin indentation) and attention, how does the nervous system dissociate between a strong-unattended and a weak-attended stimulus [45]? This ambiguity problem suggests that feature selection may rely on additional neural mechanisms that do not interfere with codes representing the sensory characteristics of stimuli, such as temporal correlation codes (see [46]–[48] for alternative explanations on visual stimuli with different contrasts). Indeed, our dataset revealed that attention enhanced spike-synchrony when it matched the preferred feature of neurons and decreased r_sc_ when it was directed away from vision. This pattern of effects suggests that selective attention implements multiple mechanisms to mediate feature selection. We posit that attention operates by suppressing the background and “correlated” population noise while enhancing the synchronous activity across the neural cohort encoding the relevant features of sensory stimuli. We postulate that these mechanisms may operate in parallel and in concert, with suppression of correlated noise underlying enhancements in focused attention (i.e., spatial attention in vision or somatotopic attention in touch) as suggested by Mitchell and colleagues [12].

**Figure 8:**
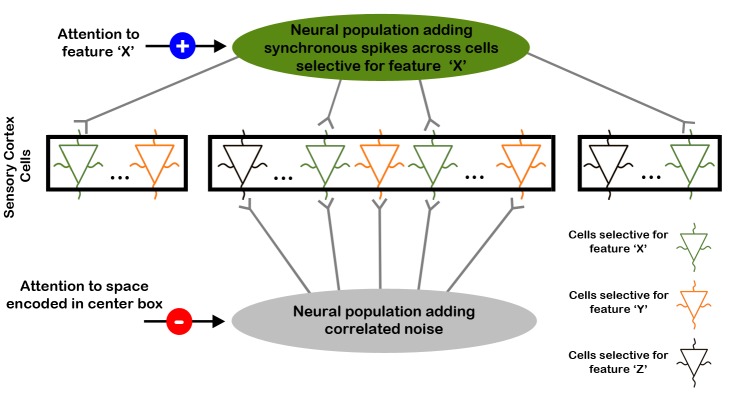
An attention model based on temporal correlation codes. The figure shows an illustration of a network model composed of three neural populations that aim to explain the attention effects on r_sc_ and spike-synchrony observed in our study. The middle portion of the figure depicts a subset of cells in sensory cortex, which have selectivity to different stimulus features (depicted by their color) and different spatial or somatotopic RF properties (as shown by rectangles encasing them). The green ellipse represents a neural population that is selective for the same stimulus features as green neurons in sensory cortex (i.e., feature “X”). This neural population causes synchronous spikes in green colored sensory cells regardless of their spatial or somatotopic RF. The gray ellipse is a neural population that has the same spatial or somatotopic RF properties as neurons inside the center rectangle but it is not selective for a stimulus feature. This neural population is responsible for adding “correlated noise” between the neurons inside the center rectangle. The orange and black colored neurons are sensory cells selective for other types of stimulus features (i.e., not selective for feature “X”).

We devised a model that may account for the attention effects observed in the spike-synchrony and r_sc_ data. Figure 8 shows a diagram of a neural population in sensory cortex (e.g., SII) that is composed of neurons selective for different stimulus features (e.g., orientation, frequency, motion) and have different spatial or somatotopic RFs (e.g., upper left visual field or digit 3 on the hand, respectively). The feature selectivity of each neuron is indicated by their color while its RF property is depicted by the box that encases it. The model proposes that all neurons selective for feature “X” receive inputs from a source that is selective for the same feature (Figure 8, upper green ellipse), regardless of whether they share the same spatial or somatotopic RF. Further, the five most inner neurons in sensory cortex receive inputs from a source that has a common RF, but it is not selective for an individual feature (Figure 8, lower gray ellipse). This neural population adds the same amount of spikes to each sensory neuron inside the center rectangle, which underlie correlated “noise.” The model contends that when attention is directed to feature “X” and to the location encoded by neurons in the center rectangle, feature-attention activates the neural population inside the green ellipse, indicated by the additive symbol inside the blue circle, which causes coincident APs in all green colored sensory neurons. In parallel, somatotopic- or spatial-attention suppresses the activity of the neural population inside the gray ellipse, indicated by the minus symbol inside the red circle, which effectively decreases the “correlated noise” added to the sensory neurons inside the center rectangle. This sequence of events leads to green colored neurons inside the center rectangle to exhibit increased spike-synchrony, and as a result, increased r_sc_ (see explanation above). This prediction is supported by our dataset, which shows increases in spike-synchrony and r_sc_ when attention is directed towards the preferred feature of cells. Importantly however, the model also predicts that neurons selective for feature “Y” (i.e., orange colored neurons) located inside the center rectangle would exhibit reduced r_sc_ without increases in spike-synchrony. This pattern is consistent with our findings of decreased r_sc_ in the absence of spike-synchrony enhancements when attention is directed away from the preferred feature of cells (see Figure 5B, middle panel, and example neurons in Figure 4A). Finally, when directing attention away from stimuli encoded by neurons inside the center rectangle, the model predicts that these cells would show increased r_sc_ in the absence of spike-synchrony enhancements. This pattern is consistent with our data showing increased r_sc_ without enhanced spike-synchrony when attention is directed towards the visual modality (Figure 5).

This model can also account for feature-attention effects in the FR of single cells, in that higher FRs are expected when attention matches the cell's feature selectivity, regardless of their RF location. In particular, the model would predict that when attention is deployed to feature “X” in the location encoded by neurons in the center rectangle, cells selective for feature “X” with RF in the flanking rectangles would exhibit increased FR as compared to cells selective for “Y” and “Z” with the same RFs. Indeed, a similar pattern of activity has been observed in MT cells in non-human primates engaged in a motion discrimination task [13], whereby higher FR activity was observed in cells whose preferred motion direction matched the attended direction regardless of whether spatial attention was directed to the cells' RF. Although this model accounts well for the findings observed in our study and others, additional experiments are needed to validate its intricacies by testing whether attention biases the activity of the external populations depicted in the green and gray ellipses in the predicted manner.

## Materials and Methods

### Ethics Statement

All animal surgeries were performed under sterile conditions and during anesthesia. Further, all surgical and experimental procedures were approved by the internal review board (IRB) and the animal care and use committee (ACUC) of the Johns Hopkins University. Standard operant conditioning procedures were employed, whereby each animal was rewarded with drops of water or juice for every correct response. The animal's health was monitored daily by the experimenters and approximately every 2 weeks by trained veterinarians and other staff. All procedures that might have produced pain or distress were minimized.

### Subjects

Single unit (SU) responses were obtained from the hand regions of SII from five hemispheres in three male rhesus (*Macaca mulatta*) monkeys (average weight 6.73, 5.14, and 4.5 kg). Each monkey was trained to perform a visual and tactile discrimination task.

### Tactile Stimuli

The tactile stimulus consisted of two perpendicular bars (90° apart). Each bar was independently controlled by a linear motor that controlled the stimulus bar's vertical displacement and vibrating frequency (animals 1 and 2 only). The tactile bar presented to animal 3 did not vibrate, and was controlled by two motors that rotated and indented the bar to the desired angle and amount, respectively. We restricted stimulation to the distal pads of digits 2, 3, or 4 depending on the RF of the recorded neuron. The vast majority of neurons had RFs that span multiple digits, and sometimes the entire hand [49]. For these neurons, the stimulator was placed on the distal pad that evoked the largest firing activity (i.e., the hotspot).

For animals 1 and 2 the orientation of the bars was either 45° or 135° relative to the long axis of the finger and the vibration frequency was either 10 or 40 Hz. All combinations of vibration frequencies and orientations were presented with equal likelihood. To compensate for differences in stimulus perceptibility due to differences in intensity [50], the 10-Hz vibrating stimulus was presented with amplitudes of either 150 or 300 microns, while stimuli vibrating at 40 Hz were presented with amplitudes of either 30 or 60 microns. These frequency/amplitude combinations lie along the same iso-intensity discrimination functions of humans [51]. On the basis of the similarities in the frequency/amplitude neural threshold curves between humans and rhesus macaques [52]–[54], we argued that the animal's successful performance on the frequency task was achieved by focusing on the vibrating feature of the sensory stimulus as opposed to its indentation amplitude. For animal 3 oriented bars with angles ranging from 0° to 157.5°, relative to the long axis of the finger, were presented. All stimuli were presented for a period of 500 ms with an on/off ramp of 25 ms.

### Experimental Paradigm (Animals 1 and 2)

#### Cueing stimuli

A visual cue was used to signal the animal as to which task to perform on every trial. A green triangle instructed the animal to perform the tactile-orientation task, while a red circle to perform the tactile-frequency task. Finally, a blue square indicated the animal to perform the visual task (see below for details). Cues were presented in the center of a monitor placed in front of the animal for the entire trial duration (3,484 ms) with size of 2.04°. During the experimental sessions the second monkey did not perform the tactile-orientation task because its hit-rate during training never exceeded 60% after about 4 months of training in that condition.

#### Experimental set up and task sequence

The animal was seated in a comfortable chair with the head restrained. The animal's hands were supinated and restrained throughout the recording session. The sequence of events in a typical trial experienced by animals 1 and 2 is illustrated in Figure 1A. A trial commenced with the presentation of a visual cue, which was followed by blank period of 950 ms. If the animal successfully maintained fixation the tactile stimulus was presented. Following 300 ms after the onset of the tactile stimulus (500 ms for monkey 2) a response cue, in the form of two white circles presented on the left and right of the visual cue (each 2.04° in diameter and equi-luminant), was presented. The response cue was delayed in the second monkey to discourage it from making incorrect responses. The animal made a saccade to one of the circles depending on the task. During attend-orientation trials, a 45° oriented stimulus required a saccade to the right circle, while a 135° oriented stimulus required a saccade to the left circle. During attend-frequency trials, a 10 Hz stimulus required a saccade to the left circle, while a 40 Hz stimulus required a saccade to the right circle. These response contingencies were counterbalanced across animals. Note that for attend-orientation trials the animal was trained to ignore the vibrating feature of the stimulus, and *vice versa* for the attend-frequency trials.

For the attend-visual trials, the same sequence of events was implemented, but the two bilateral circles were presented with different luminance levels. In this case, the animal was required to ignore the tactile stimulus and make a saccade to the brighter circle. The two visual circles were presented for 1,000 ms. The discrimination difficulty was adapted using an ongoing staircase method based on the animal's performance. The difficulty increased (i.e., the luminance difference decreased, using a logarithmic scale) following three successive correct trials, and decreased after each error.

If the animal broke fixation prior to the presentation of the white circles, the trial was aborted and repeated (this occurred on <5% of trials). The animal was rewarded with a small drop of juice or water after every correct response. All visual stimuli were presented on a Samsung SyncMaster 740b 17″ LCD monitor, on a black background with a 60 Hz refresh rate. Eye position was monitored with a PC-60 ViewPoint EyeTracker (Arrington Research). An experimental block contained 60 trials for monkey 1 and 40 trials for monkey 2. The inter-trial-interval was set to 2,034 ms.

### Experimental Paradigm (Animal 3)

The animal was seated in a comfortable chair with the head restrained. The animal's hands were supinated and restrained throughout the recording session. The sequence of events in a typical trial for this animal is illustrated in Figure 1B. A trial began with an oriented bar indented on one of the animal's distal fingerpad for 500 ms (0° to 157.5°, in steps of 22.5°). After a delay period of 900 ms a second oriented bar was indented on the same fingerpad and with the same duration. The second stimulus had the same or an orthogonal orientation (i.e., 90° difference) to the first stimulus. In the attend orientation trials, the animal pressed a foot switch in the forward or backward direction if the stimuli had the same or different orientation, respectively. In attend visual trials, the animal experienced the same tactile stimulation, but it was trained to press the foot switch when a white square (2° visual angle), which was continuously presented on the screen, was dimmed. A drop of liquid was given for every correct trial. This animal performed the tactile and visual trials on separate blocks, and this was cued by changing the pattern on the screen from an illuminated square (visual task) to a blank screen (tactile task).

### Characterization of Neural Selectivity for Tactile Features

Separate blocks of trials were run to characterize a neuron's orientation and frequency selectivity to tactile features. During these trials the animal sat quietly while receiving drops of water at random intervals. Trials within each block were randomly presented. Tactile stimuli in both of these blocks were presented for 500 ms with an on/off ramp of 25 ms, and the inter-stimulus-interval set to 500 ms.

#### Orientation selectivity protocol

Eight orientation stimuli ranging from 0° to 157.5° in steps of 22.5° were presented. Each condition was randomly presented eight times.

#### Frequency selectivity protocol

Six vibrating stimuli (10, 20, 40, 60, 80, and 100 Hz) were presented. Each frequency stimulus was randomly presented with six different amplitudes. The amplitudes were different across frequencies with 10 and 20 Hz stimuli presented with amplitudes of 300, 150, 15, 10, 6, and 1 micron, while vibrating stimuli at 40, 60, 80, and 100 Hz were presented with amplitudes of 100, 60, 30, 10, 5, and 0.5 microns. Each stimulus was presented eight times. This selectivity protocol was only conducted in animals 1 and 2.

### Neurophysiology

#### Recordings

Standard neurophysiological techniques were used to collect the data in all animals. In animals 1 and 2 data were recorded from up to four separate extracellular microelectrodes (2 to 7 MΩ, Tungsten FHC Inc) driven by a custom-built microdrive system that had electrodes linearly aligned and spaced 584 µm apart. Animal's 3 data were recorded using a Reitboeck seven channel microdrive system [55] that had electrodes linearly aligned and spaced ∼400 µm apart. Both arrays were not chronically implanted, thus they were mounted on the animal's skull every recording day. Further, unlike most chronically implanted array systems, these systems allowed us to independently vary the depth of each electrode with micrometer precision. Positioning along the anterior/posterior and medio/lateral axes was set on each recording day with a 2-D coordinate positioner that provided precision at the micron level. This positioner device also allowed us to vary the angular direction of the array when penetrating the brain with ∼5° precision. The recording chamber (19 mm diameter) was centered over the Horsley-Clarke coordinates: anterior = 6; lateral = 28. We tested a neuron's cutaneous sensitivity by brushing or indenting the glabrous and hairy skin using blunt probes. Only neurons with clear cutaneous responses in the distal finger pads of digits 2, 3, and 4 were included in the experiment. SU were isolated using a time/amplitude multi-template matching algorithm [56], and only one neuron per electrode was recorded at a time. The shape and timing information of each AP was stored, and additional SU isolation analyses were performed offline to ensure that SU activity was well isolated (see Methods S1). Unfortunately, because of technical limitations the shape of the APs could not be stored in animal 3. However, only APs that displayed unique template shapes during recordings, as judged by two experimenters, were included in the analyses.

### Analyses

A total of 297 neurons were recorded from all animals. Neurons with mean rate <5 Hz across all attention blocks were discarded and only neurons that had at least eight valid trials per condition were analyzed. Further, only neurons that contained a full balanced dataset of experimental conditions (i.e., all feature characterization and attention protocols within each experiment) were included in the analyses. This led to the exclusion of 92 neurons, leaving 205 neurons (animal 1 = 89, animal 2 = 40, and animal 3 = 76) and 122 neural pairs retained for analyses. An important requirement of our experiment was to record neural activity from multiple well-isolated single neurons with specific tuning characteristics at the same time. This precluded us from recording simultaneous activity from a large sample of neural pairs, as previous studies have done using chronically implanted micro-electrode arrays (e.g., [9]–[11]).

Our primary objective was to investigate how feature-based attention modulates activity of feature selective neurons. To this end, most analyses were performed on neurons that had preference for a particular tactile feature. This resulted in the analysis of 128 single neurons (65 orientation-selective only, 30 frequency-selective only, 39 selective for both types of tactile-features) and 57 neural pairs. Neurons that were selective for both types of tactile-features were discarded from all analyses of attention effects.

#### Analyses of mean firing rate

All AP trains were aligned to the onset of the tactile stimulus. To characterize a neuron's feature selectivity, the mean FR within a 200 ms window (centered on the average peak) was used for statistical analyses. If the peak occurred before 100 ms, then the mean FR was calculated from 0 ms onwards. The FR between the best-preferred and least-preferred stimuli was submitted to a two-sample t-test, and a neuron was classified feature selective if the *p*-value was below 0.05 (see [11] for a similar analysis).

#### Analyses of spike-synchrony

Spike-synchrony was characterized using a spike-synchrony counting method (SSCM) that computed the number of times spikes from two neurons were within ±2 ms of each other. This procedure was performed for each time bin in all trials, and it is similar to the method employed by [2], see below:
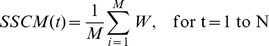
(1)

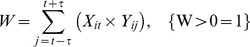
(2)where M is the number of trials, *n* = the number of bins in each spike train (1 ms bins), X and Y represent the spike trains (composed of binary values) for each neuron in the neural pair, and τ is the time lag, which was set to 2 ms. The variable “I” indicates the trial number, while “j” indicates the time bin for the second neuron composing the neural pair. Note that W was set to “1” whenever it was greater than 0. If W is not constrained, then summing across “t” results in the same value as integrating the area under the CCG across τ. The SSCM procedure has the advantage over the CCG in that it maintains the temporal structure of the spike-synchrony events, thus allowing us to assess attention effects across time, instead of the mean coincident spikes across the entire spike-train. In essence, the SSCM represents an instantaneous CCG.

We corrected the SSCM of each attention condition for effects due to common spike-rate modulations across neurons using the jitter method devised in [32]. Briefly, we divided each neuron spike train into bins of 50 ms, starting with the stimulus onset. For each spike in a trial, a new spike time was chosen randomly from the all possible times in the same jitter bin. We used a 50 ms jitter bin window as suggested by [29]. This method was repeated 5,000 times to derive a surrogate spike-synchrony distribution for each attention condition. The average surrogate data was then subtracted from the raw spike-synchrony.

Spike-synchrony attention effects were only assessed if the observed spike-synchrony rates were significantly different from derived by the jitter method. Only neural pairs whose jitter-corrected spike-synchrony was statistically significantly greater than zero for at least 100 ms (*p*-value level of 0.05) in at least one attention condition were analyzed for attention effects. Note that in all figures and analyses, the spike-synchrony derived from the jitter correction method was subtracted from the observed spike-synchrony. This is the reason for why certain figures have spike-synchrony values below zero. Further, we ensured that the each neuron's average FR remained largely stationary across trials. This was done by first sorting the mean FR of a cell across trials and fitting a quadratic function. Trials from the tail-ends were deleted until the analysis produced a non-significant fit (*p*<0.05). A quadratic function was used because visual inspection revealed that changes in FR across trials were best fitted by this function instead of a linear function. Since all experimental conditions were uniformly randomized, a negative or positive slope of the sorted trials would be indicative of cell loss or inclusion of APs from nearby cells, respectively. Importantly, in all analyses the experimenter and algorithms were blind as to which attention condition the deleted or accepted trials belonged to.

#### Analyses of spike-count correlations

r_sc_ were estimated by computing the Pearson correlation coefficient (CC) of the mean FRs between two neurons across trials. r_sc_ measures the trial-to-trial variability across two neurons to a repeated stimulus (Equation 3).
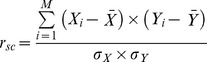
(3)where M is the number of trials, and X_i_ and Y_i_ represent the mean-rates for the “ith” trial of each neuron in the neural pair. σ_x_ and σ_y_ are the standard deviation of the mean-rates across trials for each neuron. Because these types of computations are susceptible to long-term fluctuations in FR [12], we first sorted the spike-rates of each neuron across trials and performed a linear detrending analysis. The r_sc_ values were transformed to Z-scores, using the Fisher's r-to-z method, before statistical testing
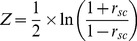
(4)


#### Statistical analysis of attention effects on FRs and spike-synchrony

Beginning with the onset of the tactile stimulus, the average activity within a 50 ms window was calculated and an ANOVA test with conditions of *attention* (attention directed to orientation, frequency, and visual) as the main factor was computed. If the data were not normally distributed we used appropriate non-parametric tests (e.g., Mann-Whitney U-test or Kruskal-Wallis). This procedure was repeated every 50 ms until the offset of the stimulus. A statistically significant effect was determined to be present if the test revealed a consecutive *p*-value <0.05 for at least 100 ms. However, a statistical effect was nullified if the direction of the attention effect reversed in future time intervals. This analysis procedure was employed because the instantaneous FR profiles of SII neurons were extremely heterogeneous (see Figure S1). Note that a 100 ms timeframe is twice as a long as those used by previous studies [2]. Because animals 2 and 3 did not perform the orientation and frequency tasks, respectively, missing values were treated using the missing data imputation method applied by the SPSS statistical package.

#### Statistical analysis of attention effects on r_sc_

Effects of attention on r_sc_ were identified by performing ANOVAs with factors of *attention* (attention directed to orientation, frequency, and visual) using the Fisher's z values as the dependent measure. Post hoc attention effects were computed using Student's *t* tests. Effects of attention on neural populations were determined using chi-square statistics. Similar to the analyses of attention effects on spike-synchrony, missing values were treated using the missing data imputation method by SPSS.

## Supporting Information

Figure S1 **Neural response heterogeneity in SII cortex.** This figure illustrates the instantaneous FR profiles of 16 neurons in SII cortex. All neurons are aligned to the onset of the tactile stimulus (t = 0).
(TIF)

Figure S2 **Attention effects on the FR.** This figure shows the FR profile of example neurons in all animals selective for frequency and orientation tactile features. Attention to frequency, orientation, and vision are represented in red, green, and blue traces, respectively. Graphs are aligned to the onset of the tactile stimulus (t = 0). The graph shows greater FRs when attention is biased towards the preferred feature of the cell compared to vision.
(TIF)

Figure S3 **Attention effects on spike-synchrony.** (A) This figure shows the effects of attention on jitter-corrected spike-synchrony for example neural pairs selective for frequency and orientation features in all animals. Attention to orientation, frequency, and vision are represented in green, red, and blue traces, respectively. Graphs are aligned to the onset of the tactile stimulus (t = 0).
(TIF)

Figure S4 **Feature attention effects as a function of neurons' feature selectivity index.** These graphs illustrate a null relationship between FAI and neurons' feature selectivity for the FRs, spike-synchrony, and r_sc_ data. The x-axis on each graph represents the difference between cells' preferred (highest) FMSI and the non-preferred (lowest) FMSI. The y-axis on each graph represents the FAI derived by subtracting the mean response when attention was directed away from neurons' preferred feature to the mean response when attention was directed towards cells' preferred feature, and dividing this difference by the sum of these two quantities. The left, middle, and right panel represent the FAI for the firing-rates, spike-synchrony, and rSC data, respectively. The black and gray dots represent the FAI for feature selective and non-feature selective cells. These data did not reveal a systematic relationship for any measure.
(TIF)

Figure S5 **Spike-synchrony as a function of neural distance between features-selective neural pairs.** This graph illustrates that there is no relationship between the strength of spike-synchrony and the distance between the neural pairs. Each dot represents a neural pair. Because of technical limitations we were not able to extract the depth values in 14 neural pairs. A regression analysis revealed no statistical relationship between these two measures (F(1,37) = 0.0023, *p*>0.05).
(TIF)

Figure S6 **Models of a source that modulates the correlated spiking activity between two neurons.** (A) The left panel illustrates a piecewise non-homogeneous Poisson rate function (mean FR 25 Hz), which was used to generate spike trains for the models in (B) and (C). The right panel shows the corresponding raster plots. (B) This figure is an illustration of a source that modulates the responses of two neurons by causing a temporally-coincident spike. This source is periodic, as depicted by the top black bars. The blue and red bars indicate the spikes of cell “X” and “Y,” respectively. The superimposed black bars reflect the common spikes caused by the periodic source. Note that these spikes are aligned to the top black bars. In addition, in some occasions, the number of coincident spikes varies. (C) This figure is an illustration of a periodic source that modulates the Poisson rate functions of two neurons in the same manner. The periodic source is depicted in the black sinusoid wave. The blue and red bars indicate the spikes of cell “X” and “Y,” respectively. Note that the Poisson rate function of each cell is reduced during the “down” cycle of the periodic source signal.
(TIF)

Data S1 **Spreadsheet document containing the data illustrated in** **Figures 2** **to** **7** **in the main manuscript.** The data from each figure are organized in separate worksheets. The labels for each condition are displayed on the top rows in each worksheet. The data points for each condition are arranged across rows.
(XLSX)

Methods S1 **A full description of (1) single-unit isolation and neural acceptance procedures, (2) measurements of attention in animals 2 and 3, (3) the analysis of attention effects as a function of cells' FMSI, (4) the analysis of spike-synchrony as a function of electrode distance, and (5) the simulation models of a source that modulates the correlated spiking activity between two neurons.**
(DOCX)

## Abbreviations

AP: action potential
CCG: cross-correlogram
FAI: feature attention index
FMSI: feature modality selectivity index
FR: firing rate
MT: medio-temporal
RF: receptive field
r_sc_: spike-count correlations
SII: secondary somatosensory cortex
SSCM: spike-synchrony counting method
SU: single unit
V1: primary visual cortex
V4: visual area 4
