# Supplementary figures and images for: Temporal Correlation Mechanisms and Their Role in Feature Selection: A Single-Unit Study in Primate Somatosensory Cortex
Source: PLoS Biol. 2014 Nov 25;12(11):e1002004. doi: 10.1371/journal.pbio.1002004 (PMC4244037; doi:10.1371/journal.pbio.1002004)

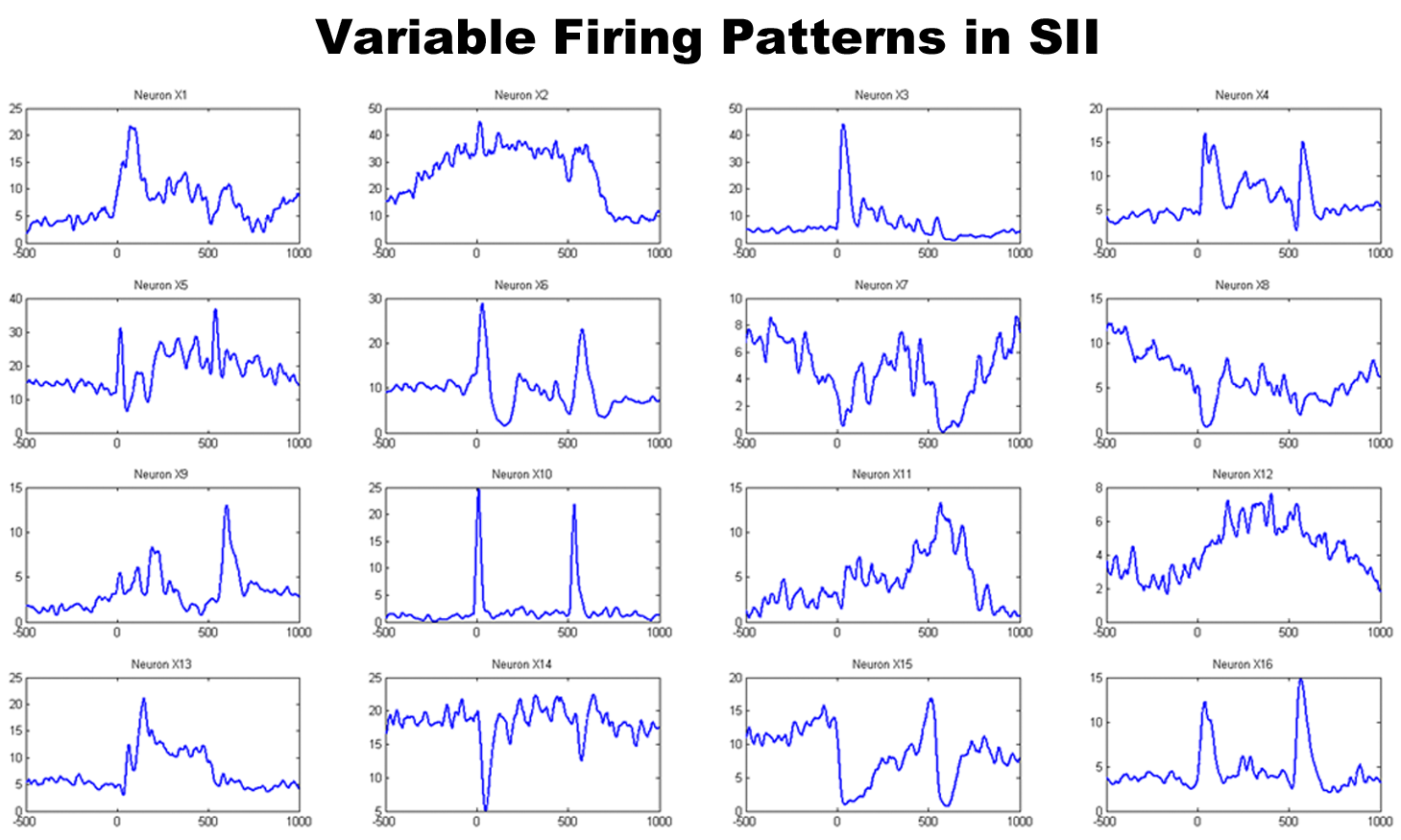

Supplement: Figure S1 — Neural response heterogeneity in SII cortex. This figure illustrates the instantaneous FR profiles of 16 neurons in SII cortex. All neurons are aligned to the onset of the tactile stimulus (t = 0). (TIF) [file pbio.1002004.s001.tif]

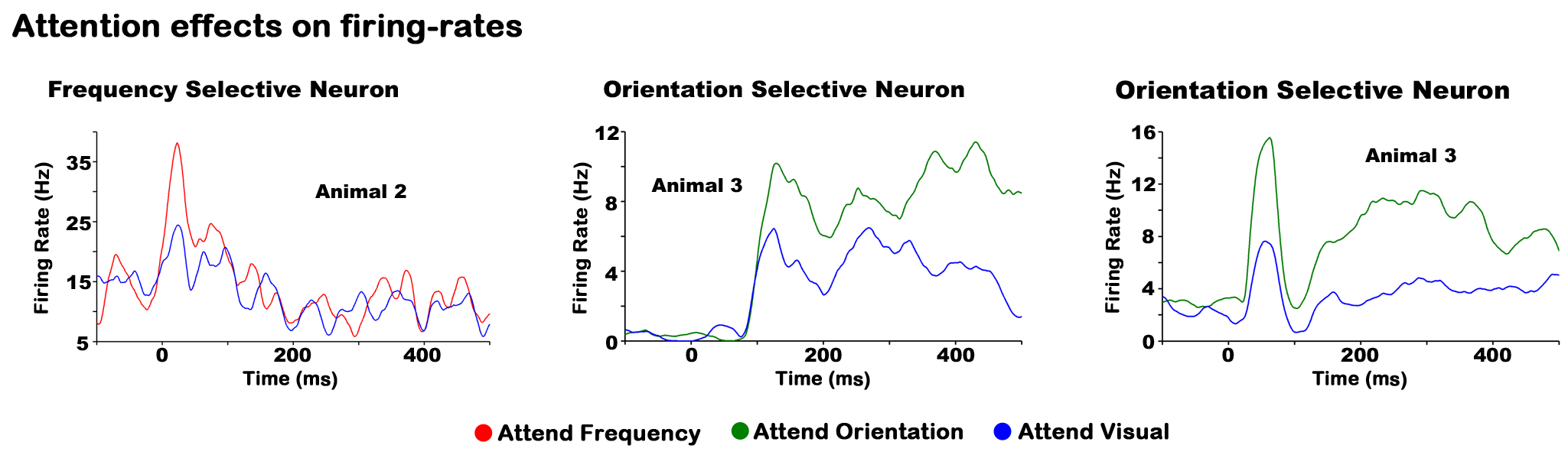

Supplement: Figure S2 — Attention effects on the FR. This figure shows the FR profile of example neurons in all animals selective for frequency and orientation tactile features. Attention to frequency, orientation, and vision are represented in red, green, and blue traces, respectively. Graphs are aligned to the onset of the tactile stimulus (t = 0). The graph shows greater FRs when attention is biased towards the preferred feature of the cell compared to vision. (TIF) [file pbio.1002004.s002.tif]

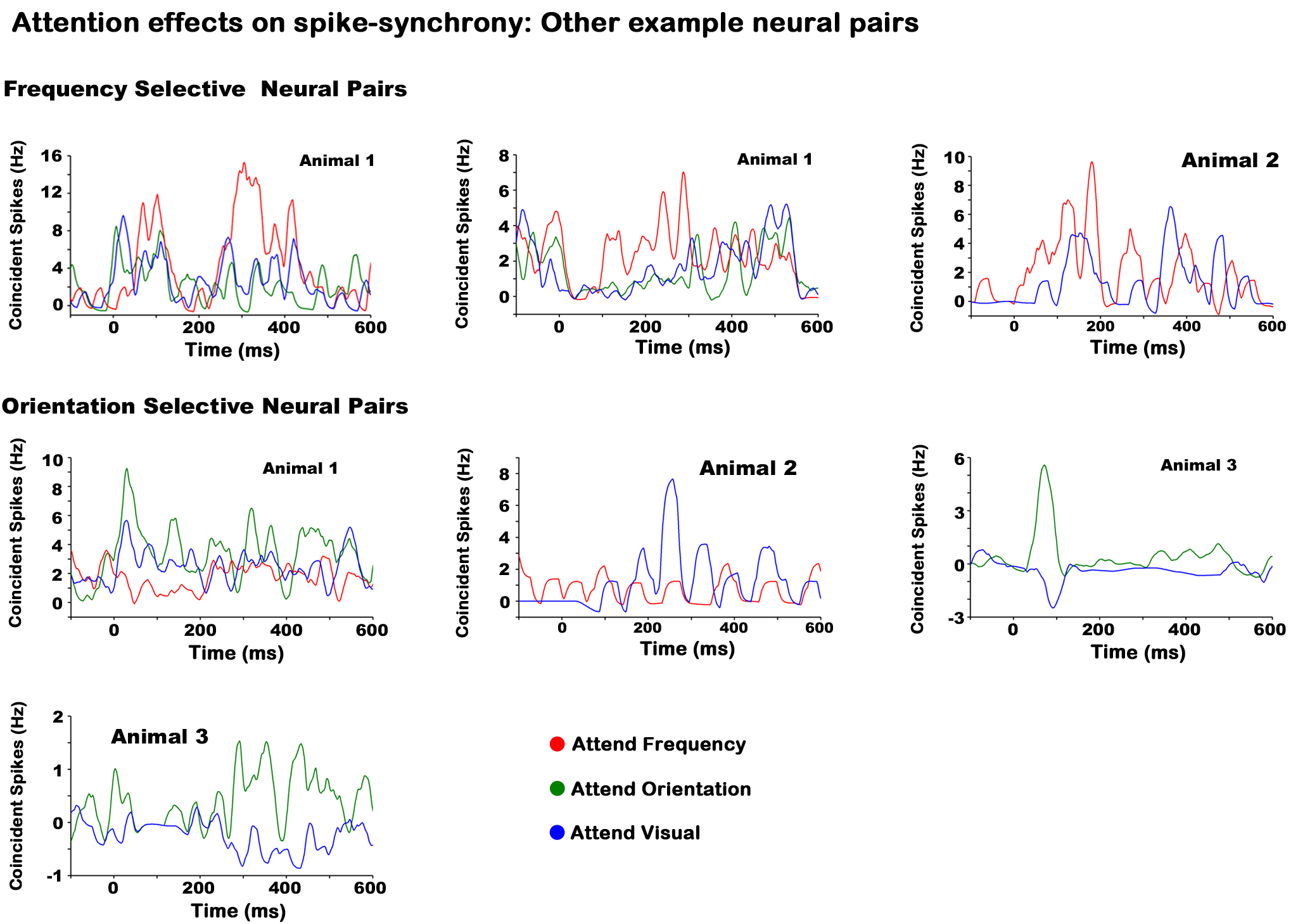

Supplement: Figure S3 — Attention effects on spike-synchrony. (A) This figure shows the effects of attention on jitter-corrected spike-synchrony for example neural pairs selective for frequency and orientation features in all animals. Attention to orientation, frequency, and vision are represented in green, red, and blue traces, respectively. Graphs are aligned to the onset of the tactile stimulus (t = 0). (TIF) [file pbio.1002004.s003.tif]

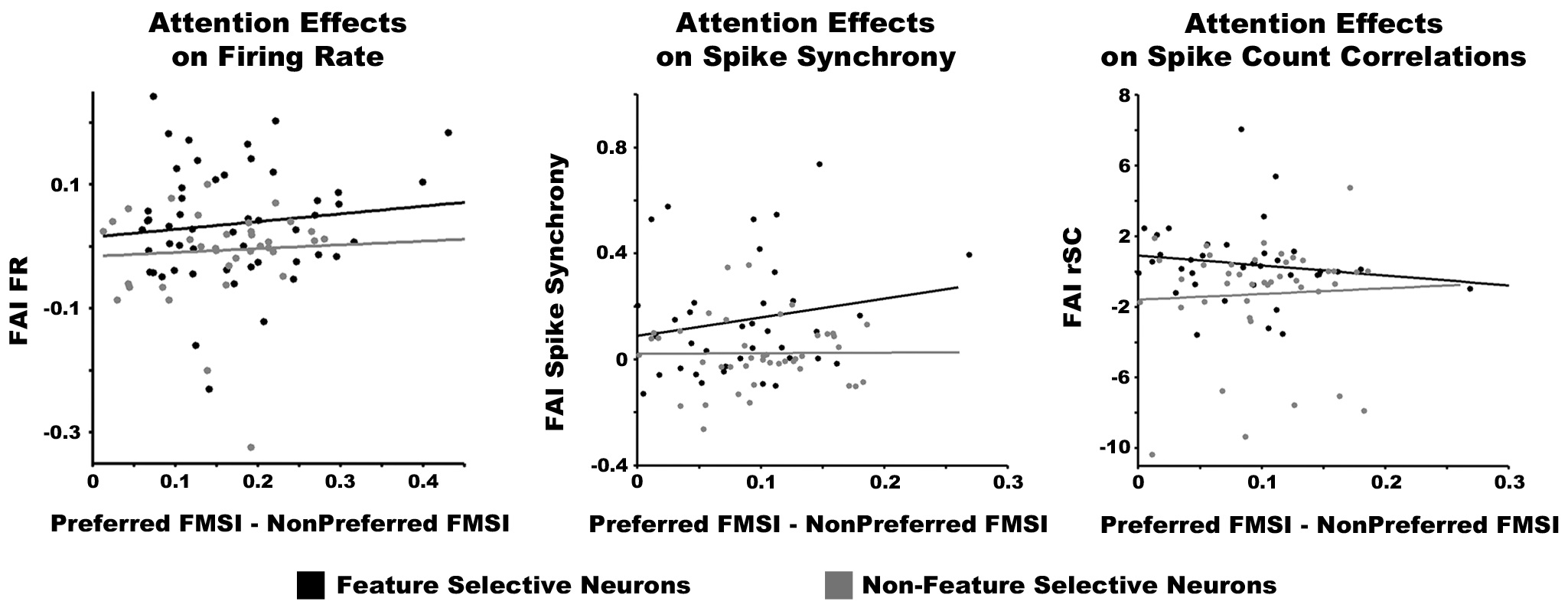

Supplement: Figure S4 — Feature attention effects as a function of neurons' feature selectivity index. These graphs illustrate a null relationship between FAI and neurons' feature selectivity for the FRs, spike-synchrony, and rsc data. The x-axis on each graph represents the difference between cells' preferred (highest) FMSI and the non-preferred (lowest) FMSI. The y-axis on each graph represents the FAI derived by subtracting the mean response when attention was directed away from neurons' preferred feature to the mean response when attention was directed towards cells' preferred feature, and dividing this difference by the sum of these two quantities. The left, middle, and right panel represent the FAI for the firing-rates, spike-synchrony, and rSC data, respectively. The black and gray dots represent the FAI for feature selective and non-feature selective cells. These data did not reveal a systematic relationship for any measure. (TIF) [file pbio.1002004.s004.tif]

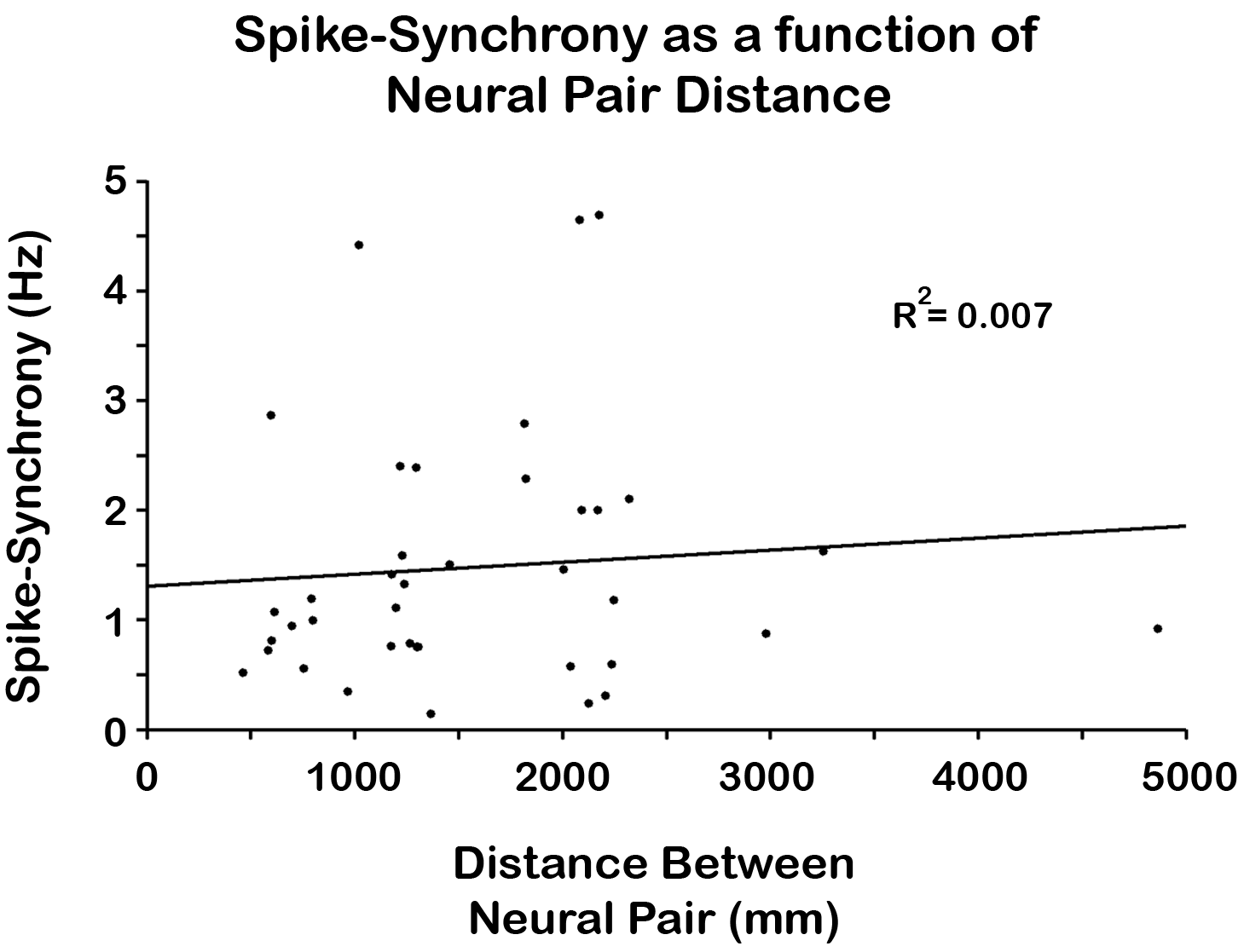

Supplement: Figure S5 — Spike-synchrony as a function of neural distance between features-selective neural pairs. This graph illustrates that there is no relationship between the strength of spike-synchrony and the distance between the neural pairs. Each dot represents a neural pair. Because of technical limitations we were not able to extract the depth values in 14 neural pairs. A regression analysis revealed no statistical relationship between these two measures (F(1,37) = 0.0023, p>0.05). (TIF) [file pbio.1002004.s005.tif]

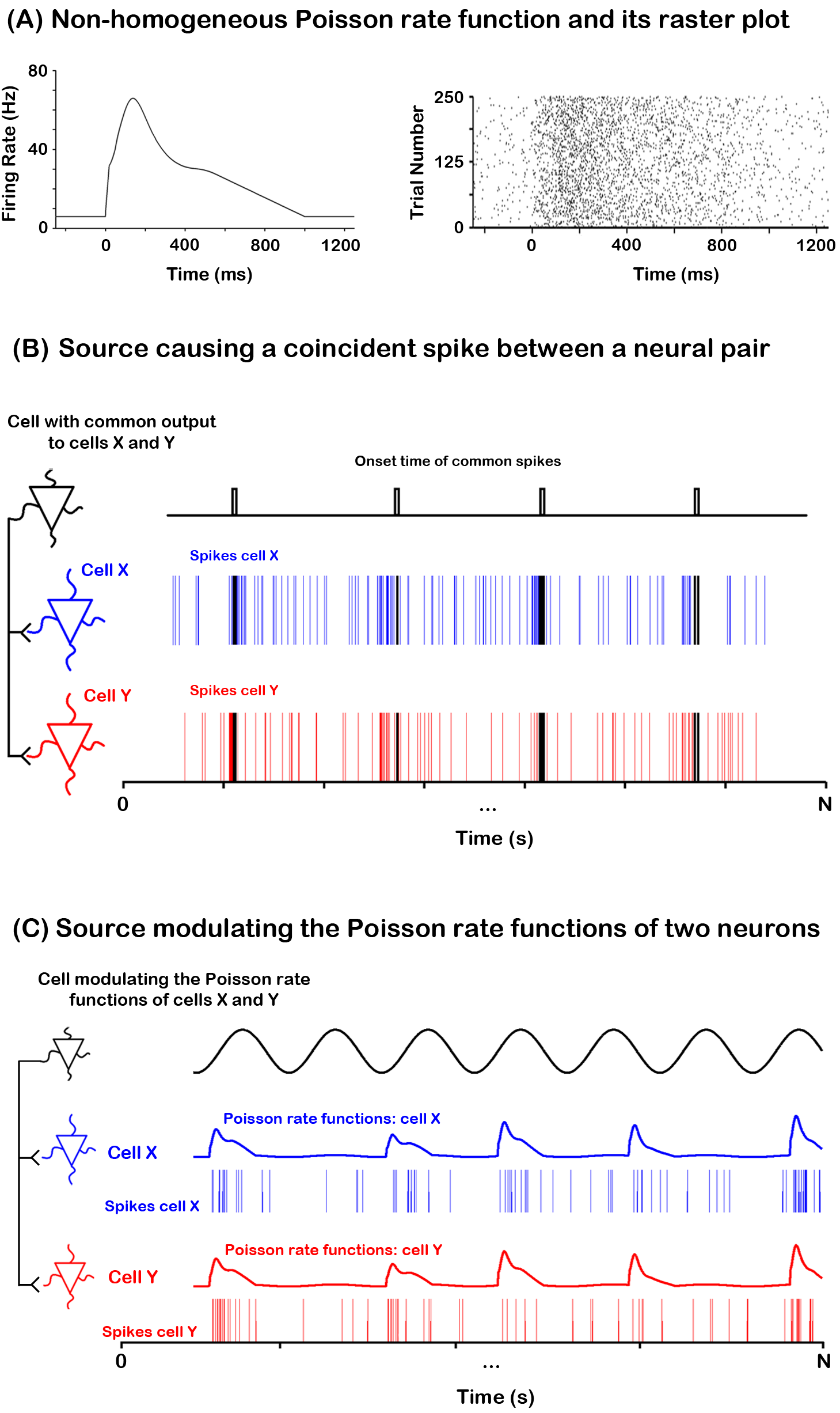

Supplement: Figure S6 — Models of a source that modulates the correlated spiking activity between two neurons. (A) The left panel illustrates a piecewise non-homogeneous Poisson rate function (mean FR 25 Hz), which was used to generate spike trains for the models in (B) and (C). The right panel shows the corresponding raster plots. (B) This figure is an illustration of a source that modulates the responses of two neurons by causing a temporally-coincident spike. This source is periodic, as depicted by the top black bars. The blue and red bars indicate the spikes of cell “X” and “Y,” respectively. The superimposed black bars reflect the common spikes caused by the periodic source. Note that these spikes are aligned to the top black bars. In addition, in some occasions, the number of coincident spikes varies. (C) This figure is an illustration of a periodic source that modulates the Poisson rate functions of two neurons in the same manner. The periodic source is depicted in the black sinusoid wave. The blue and red bars indicate the spikes of cell “X” and “Y,” respectively. Note that the Poisson rate function of each cell is reduced during the “down” cycle of the periodic source signal. (TIF) [file pbio.1002004.s006.tif]
