# Supplementary material for: Temporal Correlation Mechanisms and Their Role in Feature Selection: A Single-Unit Study in Primate Somatosensory Cortex
Source: PLoS Biol. 2014 Nov 25;12(11):e1002004. doi: 10.1371/journal.pbio.1002004 (PMC4244037; doi:10.1371/journal.pbio.1002004)
Supplement: Methods S1 — A full description of (1) single-unit isolation and neural acceptance procedures, (2) measurements of attention in animals 2 and 3, (3) the analysis of attention effects as a function of cells' FMSI, (4) the analysis of spike-synchrony as a function of electrode distance, and (5) the simulation models of a source that modulates the correlated spiking activity between two neurons. (DOCX) [file pbio.1002004.s008.docx]

**SUPPORTING INFORMATION**

**METHODS**

*SU isolation & neural acceptance:* Great care was taken to achieve a high level of single-cell isolation. We implemented several offline procedures to ensure that each cell included in the statistical analysis was well isolated. First, we sorted the mean firing rate (FR) of a cell across trials and fitted a quadratic function. Trials from the tail-ends were deleted until the fit was non-significant (p < 0.05). A quadratic function was used because visual inspection revealed that changes in FR across trials were best fitted by this instead of a linear function. Since all experimental conditions were uniformly randomized, a negative or positive slope of the sorted trials would be indicative of cell loss or inclusion of action potentials (AP) from nearby cells, respectively. Second, within each trial, AP shapes with a signal-to-noise ratio (SNR) less than 14db were deleted. Third, the shape of the AP was subjected to principal component analysis (PCA), and shapes that were more than 2 standard deviations away from the center of mass of the two most principal components (using the normalized Euclidean distance method) were deleted. Finally, the experimenter visually inspected each block of trials and manually deleted APs that were deemed outliers. This last procedure was based on the shape of the AP. Importantly, in all analyses the experimenter and algorithms were blind as to which attention condition the AP belonged to. Unfortunately, due to technical limitations the AP shapes of animal 3 were not recorded, thus steps 2, 3 and 4 were not applied to these data. However, only unique and robust AP templates, judged by two experimenters during the recording session, were included for this animal.

*Mean-rate and spike-synchrony attention effects in animals 2 and 3:* Animals 2 and 3 did not perform the orientation and frequency tasks, respectively. Therefore, attention effects were analyzed in neurons that were selective for frequency (animal 2) and orientation (animal 3) features only. Figures S2 and S3 show other examples of attention effects on FR and spike-synchrony, respectively.*­*

*Effects of attention as a function of the cells’ feature modality selectivity index:* We investigated whether the effects of attention on firing rates, spike-synchrony and rSC modulated as a function of the cells’ feature modality selectivity index (FMSI). The FMSI was calculated by taking the difference between the stimulus condition that evoked the highest and lowest response in the neuron’s tuning curve for each tactile feature separately (orientation and frequency).

*Spike-synchrony as a function of electrode distance:* We assessed whether there was a relationship between the distance between the two members of a neuron pairs distance and their spike-synchrony rates. This analysis was performed on all feature-selective neural pairs collapsed across attention conditions. Specifically, the spike-synchrony for each neural pair was sorted as a function of electrode distance and a linear regression, using electrode distance as the predictor variable, was performed. Due to technical limitations we were not able to record the positions of eight neural pairs.

*Models of a source that modulates the correlated spiking activity between two neurons.* We performed a series of numerical simulations aimed at explaining the spike-synchrony and rSC relationship observed in Figure 6a. Particularly, we developed two separate models in which a source modulates the correlated spiking activity between two neurons, whose spike trains were derived using a non-homogenous Poisson function. The resulting raster plot of the non-homogenous Poisson rate function is shown on the right side of Figure S6a. The correlated spiking activity within a neural population was produced by a source that: (1) caused a temporally-coincident spike across the population (Figure S6b), or (2) co-modulated the FR function of all neurons in the same manner (Figure S6c). The source that added concurrent action potentials to each spike train (scenario 1) or modulated the Poisson response functions of each spike train in the same manner (scenario 2) was a 2.5 Hz periodic signal. On average, the source in scenario 1 added 5 spikes every 400 ms (0 – 10 spikes uniformly varied). In scenario two, the non-homogenous Poisson function was multiplied by the modulating signal (i.e. the source), with an amplitude from 0.5 to 1. A. We chose a 2.5 Hz signal based on the findings by Mitchell et al (2009), which showed that most of the correlated spiking activity across a neural population is captured in the ongoing oscillating activity between 0 and 5 Hz. The blue and red bars indicate the spikes of cell ‘X’ and ‘Y’, respectively. The superimposed black bars reflect the common spikes caused by the periodic source. Note that the Poisson rate functions in scenario 2 are reduced during the ‘down’ cycle of the periodic source signal.
